# Efficacy of foliar application of *Chlorella vulgaris* extract on chemical composition and biological activities of the essential oil of spearmint (*Mentha spicata* L.)

**DOI:** 10.1016/j.heliyon.2024.e40531

**Published:** 2024-11-22

**Authors:** Fatemeh Jamshidi-Kia, Keramatolah Saeidi, Zahra Lorigooini, Bahram Hosseinzadeh Samani

**Affiliations:** a Department of Horticulture Science, Faculty of Agriculture, Shahrekord University, Iran; b Medical Plants Research Center, Basic Health Sciences Institute, Shahrekord University of Medical Sciences, Shahrekord, Iran; c Department of Mechanical Engineering of Biosystems, Shahrekord University, Iran

**Keywords:** *Mentha spicata*, Microalgae, Essential oil, Gas chromatography-mass spectrometry, Antioxidant activities, Antibacterial activities

## Abstract

The microalgal have an essential role in agriculture, where they are used as biofertilizers. This study aimed to determine the effect of *C. vulgaris* extract on the chemical composition and biological activities of the Essential Oil (EO) of Mentha spicata. The extract of *C. vulgaris* was prepared and applied at three different concentrations (50, 75, and 100 %). The EOs of M. spicata were analyzed by gas chromatography-mass spectrometry (GC-MS). The DPPH radical scavenging capability and Ferric Reducing Antioxidant Power (FRAP) techniques were used to assess the antioxidant activity of EOs. The antimicrobial activity of EO was evaluated using the microdilution method against *Staphylococcus aureus*. The results of GC-MS analysis of EOs identified 46 components, with Carvone (77.5–65.4 %), Limonene (10.31–6.9 %), β-elemene (1.56–0.98 %), and Caryophyllene (10.92–4.77 %) being the predominant constituents. From the highest concentration ranged from 100 % *C. vulgaris* extract to control respectively, yield and EO content ranged from 171.24 to 131.74 g/m2 and 0.34 to 0.18 %, respectively; Antioxidant activity by DPPH and FRAP methods varied from 1.56 to 4.45 mg/mL, and 405.63 to 68.68 μMFe2+/g, respectively; the Minimum Inhibitory Concentrations (MIC) ranged from 2.4 to 9.6 mg/mL in various treatments. The results indicated that the *C. vulgaris* extract significantly increased the yield, EO%, Carvone, Limonene, and antioxidant and antibacterial activities compared to the control. The extract of *C. vulgaris* showed promise as a biofertilizer to enhance the yield, chemical composition, and biological activities of *M. spicata*.

## Highlights

• Microalgae *Chlorella vulgaris* is a source of bioactive compounds.
• Foliar application *Chlorella vulgaris* extract improve growth of *Mentha spicata.*
• *Chlorella vulgaris* can potentially be used as a biofertilizer for plant growth.
• *C. vulgaris* extract has influences on the chemical composition of *M. spicata.*
• *C. vulgaris* extract has positive effects on biological activities of *M. spicata.*

## Graphical abstract

**Figure:**
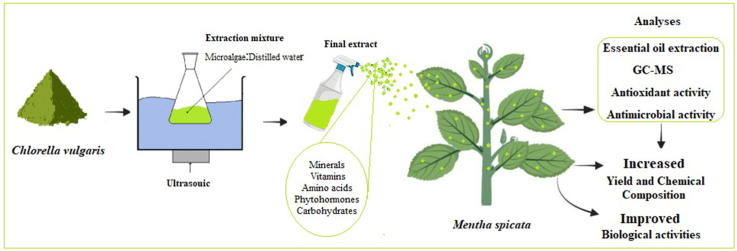

## 1 Introduction

*Mentha spicata*, commonly known as spearmint, is a member of the Lamiaceae family. It is cultivated globally for its medicinal properties, fragrance, and economic importance [1]. Spearmint was used in traditional medicine for common colds, coughs, flatulence, vomiting, indigestion, respiratory and digestive disorders, and loss of appetite. The essential oil of spearmint has been widely used as a source of essential oil for flavouring agents, antimicrobial activity, antioxidant, analgesic, anti-inflammatory, and antipyretic purposes [[2], [3], [4], [5]].

In recent years, the cultivation of medicinal plants has increased significantly to meet the growing demand for their medicinal or aromatic properties in various industries, including nutraceuticals, pharmaceuticals, cosmetics, etc. Applying chemical fertilizers is a widespread approach to achieving optimal crop yield and economic viability in plant production [[6], [7], [8], [9], [10]]. However, the inappropriate use of chemical fertilizers can have significant implications for environmental and human health. Long-term studies indicate that excessive use of chemical fertilizers diminishes plant performance due to soil acidification, reduced biological activity, and the degradation of soil physical properties. Moreover, improper management of chemical fertilizers has led to severe and often irreversible health and economic consequences. This issue is especially critical in the cultivation of medicinal plants, where the presence of chemical residues in the extracted active ingredients can compromise their quality or render them unusable [[11], [12], [13]].

Therefore, it is a growing necessity to adopt environmentally friendly methods for sustainable crop cultivation, minimizing ecological harm. This involves reducing or replacing chemical inputs, particularly fertilizers and pesticides, with natural or biological alternatives [[14], [15], [16], [17]]. Among the available alternatives, biofertilizers, especially those derived from microalgae, have garnered significant interest. Microalgae have shown remarkable capability in improving soil fertility and promoting plant growth [[18], [19], [20], [21]]. Numerous studies have shown that the use of microalgae in soil or as a foliar application leads to varying positives for plants, including increased yield, improved resistance to pests and diseases, and improved quality of chemical composition of secondary metabolites of medicinal plants, germination, and root growth [[22], [23], [24], [25]]. The *Chlorella vulgaris* strains is a group of green algae (Chlorophyceae) that exhibit fast growth and are capable of growing in various habitats, such as seawater, freshwater, and soil. *C. vulgaris* is a source of various nutrients and bioactive compounds, including essential nutrients, proteins, minerals, pigments, lipids, vitamins, and antioxidants. Several studies have found positive responses to the application of *C. vulgaris* to plants, including improved quality and plant growth [[26], [27], [28], [29]]. The objective of the present work study to evaluate the potential of the foliar application of *C. vulgaris* extract for improving crop yield, chemical composition, and antioxidant and antibacterial activities of the essential oil of *M. spicata*.

## 2 Material and methods

### 2.1 Experimental conditions

This study was carried out in 2022–2023 at the Research Farm of Shahrekord University in Iran, using a randomized complete block design with three replications (Longitude 50° 49° East, Latitude 32° 21° North, and an altitude of 2050 m above sea level, situated in Shahrekord, Chaharmahal and Bakhtiari province, Iran). The study was set up as a randomized block design comprising 15 blocks. Samples were gathered randomly using a soil auger from ten different points at depths ranging from 0 cm to 30 cm to perform the soil analysis. The collected samples underwent analysis using specified methods (Table 1) [30].

**Table 1:** Physical and chemical analysis of the soil.

Property	Soil
Texture	Clay loam
EC (ds/m)	1.451
pH	7.89
N (%)	0.077
TNV (%)	23.5
OC (%)	0.897
B (mg.kg^−1^)	1.03
Cu (mg.kg^−1^)	0.95
Fe (mg.kg^−1^)	3.41
Mn (mg.kg^−1^)	8.19
Zn (mg.kg^−1^)	0.53
P (mg.kg^−1^)	30.4
K (mg.kg^−1^)	587

TNV: Total Neutralising Value; OC: Organic carbon.

The rhizomes of spearmint were procured from Zardband Pharmaceutical Company (national ID 10861684637). November 6, 2022, spearmint rhizomes were planted at a depth of 10 cm in plots measuring 3 × 4 m, organized into four rows and five columns. Distance of sown between row 50 cm and the distance on row 50 cm. During the cultivation period, organic agricultural practices were employed, omitting herbicides or pesticides and manually managing weeds as needed.

### 2.2 Liquid microalgae extract preparation

The dried microalgae biomass powder (*C. vulgaris*) used in the study was acquired from Surna Aquatic Food Company in Iran. The *C. vulgaris* powder was blended with distilled water in a 1:10 ratio, then exposed to ultrasonication (Digital Ultrasonic Cleaner; Model JP-4820; 200W; 42 kHz) for 7.87 min at 60 °C, and subsequently filtered using a centrifuge (PIT320 R Universal) and Whatman No. 1 filter paper. The sequence was repeated three times, and the third repetition's extract was combined. In the subsequent stage, the resultant extract underwent concentration to one-third through a rotary evaporator (IKA RV 10) at 60 °C under reduced pressure, producing a 100 % liquid algae extract stored at −20 °C for future use. The extraction conditions were obtained through the Authors' previous study [31]. The Chemical composition of the liquid extract of *C. vulgaris* is shown in Table 2.

**Table 2:** Chemical composition of liquid extract of *C. vulgaris*.

Components		Content	Components		Content
Minerals (mg/L)	N	32.43 ± 0.08	Phytohormones (nmol/L)	IAA	8.04 ± 0.05
	P	49.89 ± 0.04		ZT	1.30 ± 0.02
	K	44.77 ± 0.02		GA3	8.10 ± 0.08
	Mg	302.65 ± 0.30	Amino acids (mg/L)	Arg	0.09 ± 0.01
	Ca	14.56 ± 0.02		Pro	0.54 ± 0.01
	Zn	0.22 ± 0.03		Glu	0.91 ± 0.02
	Mn	1.48 ± 0.02		Trp	0.08 ± 0.03
	Cu	1.3 ± 0.05		Gly	0.63 ± 0.02
	Fe	2.28 ± 0.01		Met	0.22 ± 0.04
Vitamins (mg/L)	B1	8.17 ± 0.04		Ser	0.29 ± 0.01
	B2	6.36 ± 0.01		Phe	0.32 ± 0.02
	B6	13.59 ± 0.02	Total Carbohydrates (mg/L)		345.16 ± 0.90
	B7	18.48 ± 0.02			
	B12	1.43 ± 0.01			

The values are expressed as means ± standard deviation for three replicates.

### 2.3 Treatment

**Fig. 1:**
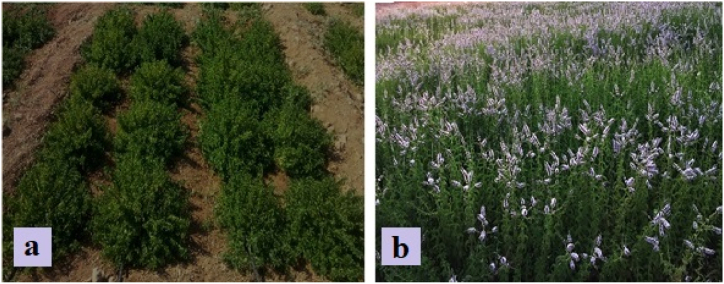
a) Vegetative stage, and b) Flowering stage of *M. spicata*.

The treatments included distilled water foliar spray (control) and foliar spray treatment with *C. vulgaris* microalgae extract at 50 %, 75 %, and 100 %. The treatments were applied during the vegetative stage, starting from 4 to 6 leaves, weekly (Once a week, Before sunrise) until full flowering, and used in three repetitions. The spearmint plant's aerial part was the subject of this research, and sampling was carried out during the flowering stage (Fig. 1(a and b)).

### 2.4 Determination of herb yield

Spearmint's aerial parts (leaves, stems, and flowers) were harvested at the full flowering stage for herb yield determination. The samples were transported to the laboratory and dried in the shade. The yield was calculated in g/m^2^ after drying.

### 2.5 Extraction of the essential oil

At the full flowering stage, the aerial parts of spearmint were harvested, dried in the shade, and ground into powder, then 50 g of the powdered aerial parts of spearmint underwent hydro-distillation for 4 h using a Clevenger apparatus to obtain their EOs, following the method outlined in the British Pharmacopoeia (1988) (British Pharmacopoeia, 1988) [32]. The obtained essential oil samples were dehydrated with anhydrous sodium sulfate and then stored at 4 °C until needed. The EO content (%) was measured by following Equation (1):

(1) $$\text{EO}\;\text{content}\;\left(\%\right)=\left[\left(W_{\text{EO}}/W_{0}\right)\right]*100$$

where, W_EO_ weight of the recovered EO (g) and W_0_: weight of the plant material (g).

### 2.6 Gas chromatography (GC) analysis

The EOs were analyzed using a Thermoquest-Finnigan Trace GC instrument with a flame ionization detector (FID). The capillary column used DB-5 (30 m × 0.25 mm ID, film thickness of 0.25 mm). The oven temperature program was 60–250 °C, increased at a rate of 5 °C/min, and finally held at 250 °C isothermally for 10 min. Helium was utilized as the carrier gas with a 1.1 mL/min flow rate, and the injector and detector temperature was set to 250 °C.

### 2.7 Gas chromatography-mass spectrometry (GC/MS) analysis

GC-MS analysis was performed using a Thermoquest Finnigan gas chromatograph equipped with the same column (DB-5, 30 m × 0.25 mm i.d., film thickness 0.25 mm), coupled with a TRACE mass ion trap detector in Manchester, UK. The oven temperature program ranged from 60 to 250 °C, increasing at a rate of 5 °C/min, and finally held at 250 °C isothermally for 10 min. Helium is the carrier gas, with an ionization voltage of 70 eV. The ion source and interface temperatures were set at 200 °C and 250 °C, respectively. The mass range covered 40–460 *m*/*z*. Chromatographic peaks were identified by comparing their Kovats retention indices with those of authentic standards. Kovats indices were calculated based on a linear interpolation of the retention time of the homologous series of n-alkanes (C7-C24, Sigma). Data were further validated by comparing the mass spectra of each constituent with those stored in the Wiley 7.0 and Adams mass spectral-retention index libraries, with NIST (NIST Chemistry WebBook) and data published in the literature [33].

### 2.8 Antioxidant activity

#### 2.8.1 Free radical scavenging activity

The antioxidant activity of the EOs was evaluated by conducting the 2,2-diphenyl-1-picrylhydroazyl (DPPH) scavenging assay. Briefly, 200 mL of a 0.1 mM DPPH radical in a methanol solution was mixed with 20 μL EO in microplates. The plates were incubated for 30 min in darkness at room temperature (25 ± 2 °C). DPPH radical inhibition was measured by measuring absorbance at 517 nm using a microplate reader (STAT FAX 2100). Antioxidant activity calculated using Equation (2):

(2) $$\text{Percent}\;\left(\%\right)\;\text{Inhibition}=\left[\left(A_{B}-A_{A}/A_{B}\right)\right]*100$$

where, A_A_ absorbance values of EOs and A_B_ absorbance values of the control (DPPH and methanol as the control no EO), and the blank consisting of only methanol (99.8 %). IC50 (mg/mL) for each sample, indicating the concentration (mg/mL) required to inhibit DPPH radical formation by 50 %, determined using Microsoft Excel software (Microsoft Excel 2016, KB4011684) [34].

#### 2.8.2 Ferric reducing antioxidant power (FRAP)

**Fig. 2:**
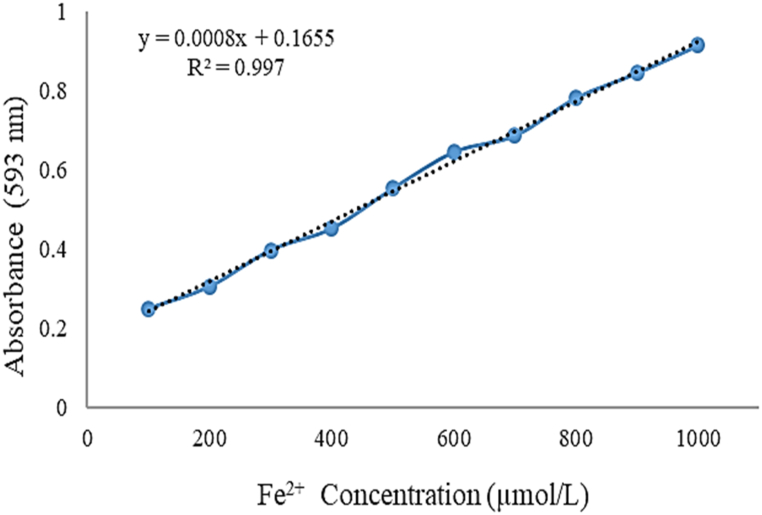
The calibration curve of Fe^2+^.

The FRAP assay was conducted as described in Benzi and Devaki (2018). Briefly, the FRAP reagent is freshly made by combining sodium acetate trihydrate (300 Mm in glacial acetic acid, pH 3.6), TPTZ (2,4,6 Tripyridyl‐S‐triazine) (10 mM in HCl (40 mM)), and FeCl3 (20 mM in distilled water) in a 10:1:1 (v/v/v). For the analysis, 3 mL of FRAP reagent was blended with 100 μL of the EOs solution (1 mg/mL in methanol) and then incubated at 37 ± 2 °C for 30 min. The absorbance read at 593 nm (UV–visible spectrophotometer, LABINDIA, UV 3000) against the blank (100 μl of methanol and 3 mL FRAP reagent). Activities of EOs derived from the standard curve of FeSO4 × 7H2O standard solutions and FRAP values expressed as μM Fe^2+^/g (Fig. 2) [35].

### 2.9 Antimicrobial activity

#### 2.9.1 Determination of minimum inhibitory concentration (MIC)

The standard microorganism strain *Staphylococcus aureus* (ATCC® 25923), was purchased from the Iranian Research Organization for Science and Technology (IROST).

The antibacterial activity of EOs was determined by using the Broth Microdilution assay. This method involves a series of dilutions of the antimicrobial agent prepared in a liquid broth medium. Then, the broth was inoculated with a standardized inoculum of the tested microorganisms. Briefly, the 96-well plates were prepared by dispensing 95 μL of broth to each well, and then 100 μL of EOs stock (dissolved in 10 % dimethyl sulfoxide (DMSO)) was added to the third row of 96-well plates. Two-fold serial dilutions were done from the third row to the end, and 5 μL of bacterial suspension (1.5 × 108 cfu mL−1) was added to the wells. Plate's first well in each row was used as a positive control (10 % DMSO solution, medium, and bacterial suspension) and the second one as a negative control (10 % DMSO solution and medium). The plates were incubated at 37 °C for 24 h. The minimum inhibitory concentration (MIC) was the lowest concentration of EOs the bacteria did not show (National Committee for Clinical Laboratory Standards, 1999) [36].

## 3 Statistical analysis

Analysis of variance (ANOVA) was performed as a randomized complete block design using SPSS 16.0 software (SPSS Inc., USA). Means of treatment compared using the least significant difference (LSD) test at a significance level of 5 %. The Hierarchical cluster analysis and Ward method were employed to assess the similarities between the chemical compositions of *M. spicata'*s EOs from different treatments.

## 4 Results and discussion

### 4.1 Herb yield and EO content

**Fig. 3:**
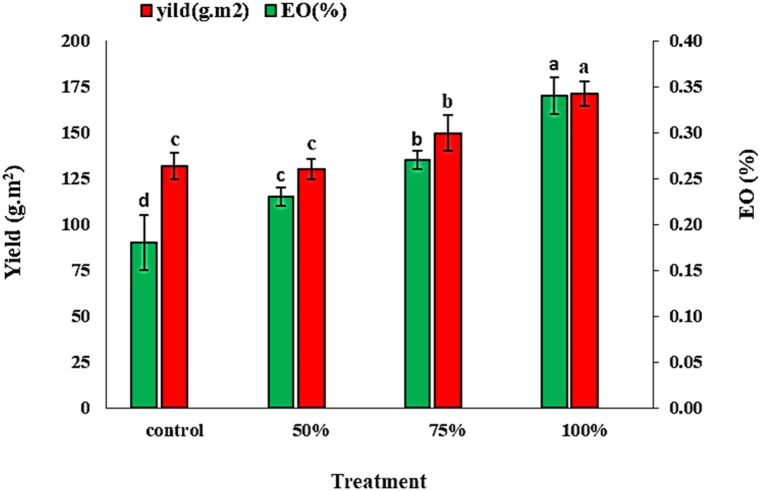
The effect of foliar application of different concentrations of *C. vulgaris* extract on herb yield and EO content of *M. spicata*. Mean values within columns followed by the same letters were not significantly different at the 5 % level according to the LSD test (P < 0.05).

The resulting analysis of the dry matter yield and EO were content showed that among the different treatments, there were significant differences (p < 0.05). Experimental results of the effects of treatment on yield and EO content are shown in Fig. 3. Results indicated that the application of *C. vulgaris* extract increased the yields of *M. spicata*. The highest herb yield and EO% (171.24 g/m^2^ and 0.34 %, respectively, were recorded for the 100 % *C. vulgaris* extract, whereas the control had the lowest 131.28 g/m^2^ and 0.18 %, respectively. Foliar application of *C. vulgaris* extract increased yield and EO%, compared to the control, and this enhancement in various treatments further improved with increasing concentrations of the extract. There are no significant differences between 50 % and control treatments in yield. Extract of *C. vulgaris* contains minerals, vitamins, amino acids, phytohormones, and total carbohydrates (Table 2) [31].

Prior research suggests that microalgae, rich in essential nutrients, can stimulate cellular metabolism and retard the aging and degradation of chlorophyll. Facilitating the absorption of nitrogen, magnesium, and iron can promote chlorophyll biosynthesis. These elements are indispensable structural components of chlorophyll. Consequently, this results in an augmentation of chlorophyll content and enhanced photosynthesis for regulating plant growth can enhance biomass production, yield, and overall quality [[37], [38], [39], [40], [41], [42]]. Microalgae extracts contain nutrients easily absorbed by leaves and stimulate metabolic reactions such as photosynthesis, respiration, and nutrient uptake; therefore, the ability of algae extract to increase growth cannot be attributed to a single type of its compounds [37,[43], [44], [45]]. Bella et al. (2021) studied the effects of foliar application of *C*. *vulgaris* extract on lettuce seedlings. Their results indicated that the extract positively influenced the seedlings, leading to increased fresh and dry weights, higher levels of chlorophyll and carotenoids, as well as improved protein and ash content in the shoots [20].

Numerous studies have indicated that microalgae *C. vulgaris* can stimulate plant growth and enhance nutrient uptake, growth, and abiotic resistance to stressors [46]. In pepper seedlings, the application of *Chlorella* extract significantly increased plant height, stem diameter, and leaf area. Additionally, treated pepper seedlings showed a marked rise in chlorophyll levels, along with significant improvements in the activities of antioxidant enzymes like superoxide dismutase, peroxidase, and catalase [47]. *Chlorella* extract has been extensively utilized as a bio-fertilizer in crops such as Chinese chives [48] and okra [49], with proven effects in boosting biomass production.

### 4.2 Chemical compositions of EOs

Analysis of the GC-MS of the EOs of *M. spicata* identified 46 components (Supplementary material). The composition of the EOs exhibited a variable pattern at different treatments. The main constituents of different treatments were Carvone (65.4–77.5 %), Limonene (6.9–10.11 %), Caryophyllene (4.77–10.92 %), and β-elemene (0.98–1.56 %) (Table 3). Other studies have reported similar compounds in the essential oil of *M. spicata*, and the major compounds were Carvone and Limonene [50]. Results indicated that the foliar application of *C. vulgaris* extract compared to the control positively impacted Carvone and Limonene but reduced the content of β-elemene and Caryophyllene.

**Table 3:** The effect of foliar application of different concentrations of *C. vulgaris* extract on chemical compositions of EOs of *M. spicata*.

Compounds	RI^a^	Treatment			
		Content (%)			
		Control	50 %	75 %	100 %
α-Pinene	939	0.27	0.32	0.29	0.27
Sabinen	979	0.15	0.15	0.18	0.12
β-Pinene	984	0.39	0.27	0.39	0.34
β-Myrcene	992	0.15	0.18	0.34	0.17
p-Cymene	1010	0.02	0.02	0.03	0.01
**Limonene**	**1036**	**6.96**	**6.90**	**10.31**	**7.64**
1,8-Cineole	1039	0.35	0.42	0.58	0.30
β-ocimene	1050	0.13	0.15	0.16	0.07
γ-Terpinene	1097	0.03	0.01	0.02	0.03
terpinolene	1100	0.04	0.04	0.05	0.07
Linalool	1105	0.09	0.07	0.07	0.09
cis-p-menth-2-en-1-o	1123	0.16	0.12	0.13	0.06
allo-Ocimene	1129	0.03	0.02	0.03	0.03
Borneol	1146	0.02	0.04	0.04	0.04
Terpinen-4-ol	1188	0.05	0.05	0.10	0.03
α-Terpieol	1197	0.01	0.01	0.01	0.01
cis-Dihydrocarvone	1201	0.02	0.01	0.02	0.08
Dihydrocarveol	1204	0.23	0.17	0.08	0.26
trans-Carveol	1207	0.84	0.79	0.49	0.18
cis-Carveol	1230	0.16	0.14	0.10	0.11
cis-3-Hexenyl isovalerate	1237	0.11	0.09	0.08	0.08
pulegone	1244	0.15	0.12	0.09	0.09
**Carvone**	**1260**	**65.40**	**69.66**	**73.04**	**77.50**
Carvone oxide	1288	0.31	0.25	0.17	0.25
Dihydrocarvyl acetate	1303	0.20	0.17	0.17	0.17
Dihydroedulan I	1309	0.27	0.23	0.08	0.16
iso-Dihydro carveol acetate	1327	0.28	0.25	0.25	0.02
δ-Elemene	1335	0.72	0.27	0.18	0.43
trans-Carveyl acetate	1342	0.13	0.12	0.10	0.03
cis-Carvyl acetate	1369	1.03	0.96	0.21	0.15
**β-Elemene**	**1403**	**1.56**	**1.28**	**1.11**	**0.98**
(Z)-Jasmone	1405	0.41	0.31	0.37	0.39
α-Gurjunene	1410	0.13	0.11	0.10	0.11
(Z)-Caryophyllene	1417	0.16	0.10	0.06	0.05
**Caryophyllene**	**1441**	**10.92**	**9.48**	**6.04**	**4.77**
α-himachalene	1448	0.32	0.27	0.14	0.18
(Z)-β-Farnesene	1460	0.09	0.05	0.05	0.06
α-Humulene	1464	0.32	0.30	0.12	0.16
α-Caryophyllene	1475	0.20	0.19	0.12	0.14
Germacrene D	1501	1.83	1.88	0.96	0.96
γ-Cadinene	1533	0.25	0.18	0.12	0.14
cis-Calamenene	1542	0.20	0.14	0.08	0.12
Caryophyllene oxide	1609	0.96	0.32	0.40	0.64
Cubenol	1638	0.41	0.48	0.36	0.29
epi-α-cadinol	1662	1.35	0.67	0.40	0.68
α-Cadinol	1677	0.24	0.50	0.06	0.13

Monoterpene Hydrocarbons		8.15	8.03	11.77	8.74
Oxygenated Monoterpenes		67.78	71.86	74.93	79.00
Sesquiterpene Hydrocarbons		16.70	14.26	9.07	8.08
Oxygenated Sesquiterpenes		2.96	1.97	1.22	1.74
Other compound		2.45	2.15	1.29	1.02

Total		98.04	98.26	98.28	98.58

RI^a^: Retention indices calculated against n-alkanes.

**Fig. 4:**
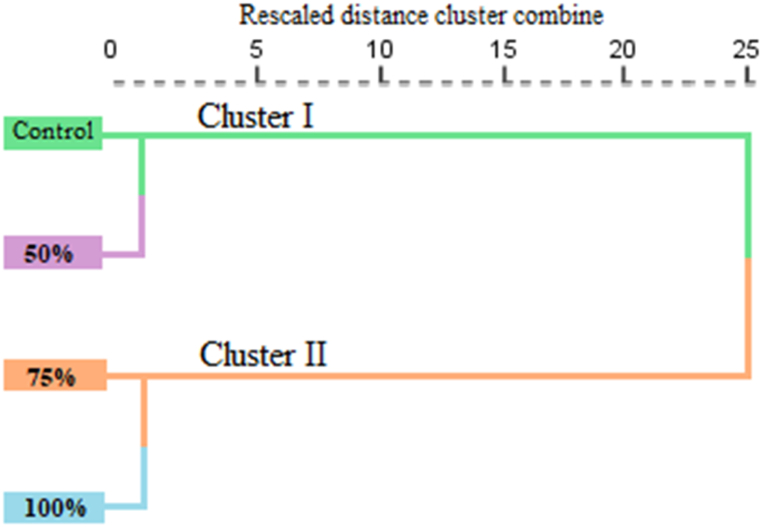
The hierarchical cluster analysis of four treatments of *M. spicata* based on EOs chemical composition using Ward clustering method.

The Cluster analysis was performed to explore the similarity among the four treatments according to their 46 chemical components of EOs. Based on the components of the groups, the results showed close similarity between the control and 50 % treatment and the treatments of 75 % and 100 % of *C. vulgaris* extract (Fig. 4). Cluster I includes 100 % and 75 % treatment; these two treatments had highly similar, suggesting that high concentrations of *C. vulgaris* extract (75%–100 %) similar chemical components of EOs. Cluster II includes control and 50 % treatment, which have chemical components similar to those of EOs. The distance between them and the first cluster is large, indicating that lower concentration of *C. vulgaris* extract (50 %) and control similar chemical components of EOs compared to the higher concentration group. The Cluster analysis showed the lower concentrations (50 %) and control are closely related, while higher concentrations of *C. vulgaris* extract (75 % and 100 %) yield different results, suggesting a threshold effect where increased concentration leads to a significant change in the chemical components of EOs.

The findings of the overall investigation suggest that foliar application of microalgae can recommended as a sustainable approach to enhance and improve the bioactive compounds of plants. Application of microalgae increased the secondary metabolites (Total flavonoid and phenolic contents, oil yield) [46,51,52]. The positive effect of *C. vulgaris* extract on the chemical compositions of *M. spicata* essential oils may attributed to the stimulation of vitamins, amino acids, and phytohormones. Plants could efficiently utilize these compounds in the extract, which could increase the biosynthesis of chemical compositions in the plants [53,54]. The role of minerals, vitamins, amino acids, phytohormones, and carbohydrates in plant secondary metabolism and biosynthesis pathways is multifaceted. The minerals such as magnesium, iron, and manganese act as enzyme cofactors. For example, magnesium is vital for ATP-dependent reactions in the shikimate pathway, while iron assists in electron transfer during terpenoid biosynthesis in the MEP/DOXP pathway [55,56].

B vitamins support amino acid biosynthesis and methyl transfers, and amino acids like phenylalanine are essential for producing secondary metabolites. Phytohormones like auxins regulate gene expression, impacting metabolic pathways, and carbohydrates contribute carbon skeletons and energy (ATP) for pathways like glycolysis, shikimate, and mevalonate, leading to the production of terpenoids, phenolics, and alkaloids. In summary, these components synergize to regulate plant metabolism by providing necessary cofactors, precursors, and signalling molecules, thus influencing the production of important secondary metabolites [[57], [58], [59]].

*C. vulgaris* extract provides essential nutrients such as nitrogen, potassium, and elements like zinc and copper, which can be vital for enzyme function and overall plant growth. Consequently, it improves the plant's physiological conditions. In addition, *C. vulgaris* extract can increase secondary metabolite production, such as essential oils. Relevant research in this field has also reported similar results that the production of secondary metabolites can improve by enhanced photosynthetic activity, which provides the ATP and NADPH required for biosynthetic pathways of metabolites [[40], [41], [42],[60], [61], [62]]. Algae extract can stimulate the biosynthesis of essential oils, either directly through its plant hormone content or indirectly by influencing the plant's endogenous hormone levels. This can be achieved by regulating enzymes involved in the production of terpenoids, a primary component of essential oils [63]. In this study, The decrease in β-elemene and Caryophyllene and the increase in Carvone and Limonene upon *C. vulgaris* extract application can be attributed to influence gene expression and enzyme activity and can stimulate enzymes like Limonene and Carvone synthase, promoting the production of monoterpenes (Carvone and Limonene) over sesquiterpenes (β-elemene and Caryophyllene) by redirecting carbon flow towards the MEP pathway [[64], [65], [66]].

Elansary et al. (2016) studied the impact of different concentrations of Ascophyllum nodosum seaweed extract on basil (*Ocimum basilicum*) and peppermint (*Mentha piperita*). They found that foliar application of the extract improved the essential oil content and composition, with Menthol and Menthone being the most abundant components in peppermint and Cavicol, Linalool, and Cineole dominating in basil Pulegone and Methofuran content decreased in peppermint [67].

**Fig. 5:**
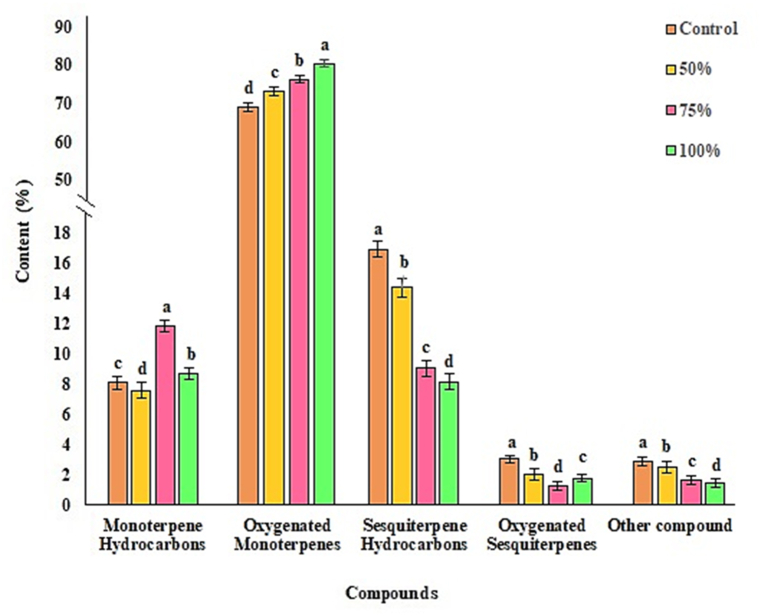
Main classes of chemical compounds of the EOs of *M. spicata* as response to foliar application *C. vulgaris* extract. Mean values within columns followed by the same letters were not significantly different at the 5 % level according to the LSD test (P < 0.05).

The results showed Oxygenated monoterpenes were the most abundant components of the EOs. The major classes were terpenes: Oxygenated monoterpenes (79.00–67.78 %) being the main subclass, followed by sesquiterpene hydrocarbons (16.7–8.08 %), Monoterpene hydrocarbons (11.77–8.03 %), Oxygenated sesquiterpenes (2.96–1.22 %), and non-terpene compounds consist 2.45 to 1.02 % of *M. spicata* Eos. The results showed that increasing extract concentrations further increased oxygenated monoterpenes in various treatments. However, it also reduced Sesquiterpene hydrocarbons' content (Fig. 5).

### 4.3 Antioxidant activity of EOs

**Fig. 6:**
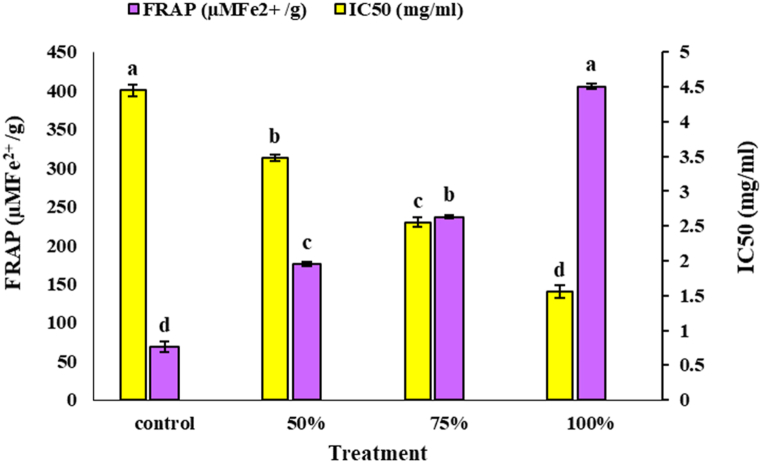
The effect of foliar application of different concentrations of *C. vulgaris* extract on antioxidant activities of EOs of *M. spicata*. Mean values within columns followed by the same letters were not significantly different at the 5 % level according to the LSD test (P < 0.05).

According to the provided results in Fig. 6, it is evident that the *C. vulgaris* extract significantly enhances the antioxidant activity of *M. spicata* EO. The IC50 values indicate the concentration required to inhibit 50 % of the free radicals, with a lower IC50 value representing higher antioxidant activity. The highest antioxidant activity was observed with 100 % *C. vulgaris* extract, which had an IC50 value of 1.56 mg/mL by the DPPH method and 405.63 μM Fe^2+^/g by the FRAP method. In contrast, the control, which did not contain *C. vulgaris* extract, showed the lowest antioxidant activity with an IC50 value of 4.45 mg/mL by the DPPH method and 68.68 μM Fe^2+^/g by the FRAP method. The EO of *M. spicata* contains various components, such as Carvone and Limonene, which are vital contributors to its antioxidant properties. These small molecules effectively capture peroxyl radicals (ROO•) and are highly susceptible to oxidation, allowing them to diffuse and neutralize radicals rapidly [68,69]. The study results support previous findings that Carvone and Limonene are significant contributors to the high antioxidant activity of *M. spicata* EO [[70], [71], [72], [73]]. The enhanced antioxidant activity observed in the EOs treated with *C. vulgaris* extract is likely due to a higher concentration of these active compounds than the control.

Moreover, several studies have confirmed that Carvone and Limonene can effectively prevent oxidative stress in cells induced by hydrogen peroxide (H_2_O_2_). They provide potential protection for normal cells against diseases associated with oxidative stress, further highlighting the valuable effects of these compounds in enhancing the antioxidant properties of *M. spicata* EO [[74], [75], [76], [77]]. These outcomes emphasize the potential of *C. vulgaris* extract in improving the efficacy of natural antioxidants in essential oils, suggesting a promising path for developing more effective antioxidant formulations.

### 4.4 Antibacterial activity of EOs

**Fig. 7:**
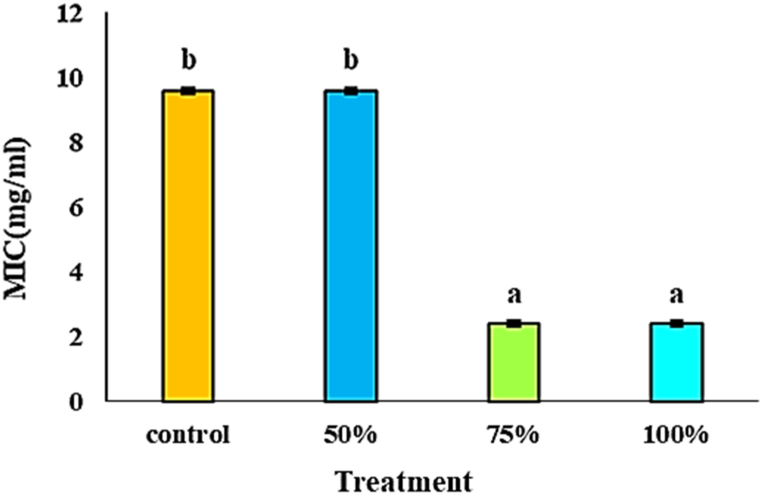
The effect of foliar application of different concentrations of *C. vulgaris* extract on antibacterial activities of EOs of *M. spicata*. Mean values within columns followed by the same letters were not significantly different at the 5 % level according to the LSD test (P < 0.05).

The MIC values of the EO of *M. spicata* against *S. aureus* are shown in Fig. 7. The Results showed significant antimicrobial activity, particularly when enhanced with *C. vulgaris* extract. The EO treated with 100 % and 75 % *C. vulgaris* extract exhibited the highest antimicrobial activity, with MIC values of 2.4 mg/mL. In contrast, the control and the 50 % *C. vulgaris* extract treatment showed the lowest activity, with 9.6 mg/mL MIC values. This heightened antimicrobial activity in the EO treated with higher concentrations of *C. vulgaris* extract can be attributed to increased monoterpenes, particularly Carvone and Limonene. These compounds are known for their potent antibacterial properties and have been previously reported as significant contributors to the antimicrobial activity of *M. spicata* EO [78,79]. In this study, heightened activity can be attributed to the higher concentrations of monoterpenes Carvone and Limonene in EO in 100 % and 75 % of *C. vulgaris* extract treatments. Previous studies reported that the monoterpenes of essential oils exhibited superior antimicrobial activity [80].

The antimicrobial mechanism of action for EOs involves several biochemical and structural disruptions within bacterial cells. Nonpolar components of EOs could disrupt bacterial structures, including the protein pumps in the bacterial membrane. This disruption alters the membrane's permeability to bacterial contents and ions, leading to cell death. Other mechanisms, such as protein denaturation and the inactivation of bacterial enzymes, also contribute to the antimicrobial efficacy. Due to the wide variety of molecules present in the EO, the antimicrobial activity of the EO cannot be attributed to a single mechanism. Different biochemical and structural mechanisms are involved on the cell surface and at multiple sites within the cell. The chemical modifications of the cell membrane, cytoplasm, enzymes, and proteins are part of these mechanisms [[81], [82], [83]]. These findings suggest that *C. vulgaris* extract could be valuable to EOs for developing more potent antimicrobial agents.

## 5 Conclusion

This research explored the foliar application of *C. vulgaris* on the chemical composition and biological activities of the essential oil of spearmint. This study demonstrates that foliar application of *C. vulgaris* extract improves spearmint yield and essential oil's chemical composition and boosts its biological activities, including antioxidant and antimicrobial properties. Specifically, applying 100 % and 75 % *C. vulgaris* extract resulted in the highest antioxidant and antimicrobial activities, attributed to increased concentrations of monoterpenes such as Carvone and Limonene. This work underscores the foliar application of *C. vulgaris* extract as a practical approach to increasing the growth of *M. spicata.* Additionally, essential oils derived from algae-treated plants are richer in bioactive compounds, with higher value and superior performance in pharmaceuticals, cosmetics, and food applications. The results show that *C. vulgaris* holds great potential to be an environmentally friendly bio-based fertilizer in sustainable agriculture to improve quality and medicinal plant growth. It can be used as a source of biologically active compounds and nutraceuticals and is exploited for significant commercial applications. Future studies should focus on the possibility of applying *C. vulgaris* extract to enhance the production of other secondary metabolites in medicinal plants. This sustainable approach could provide innovative solutions to help agricultural challenges address agricultural challenges, such as improving crop yield and resilience while reducing reliance on synthetic fertilizers.

## CRediT authorship contribution statement

**Fatemeh Jamshidi-Kia:** Writing – original draft, Validation, Methodology, Investigation. **Keramatolah Saeidi:** Writing – review & editing, Validation, Supervision, Conceptualization. **Zahra Lorigooini:** Writing – review & editing, Validation, Supervision. **Bahram Hosseinzadeh Samani:** Writing – review & editing, Software, Conceptualization.

## Data availability

All the data used in the study is included in the manuscript.

## Funding

This research did not receive any specific grant from funding agencies in the public, commercial, or not-for-profit sectors.

## Declaration of competing interest

The authors declare that they have no known competing financial interests or personal relationships that could have appeared to influence the work reported in this paper.
