# Supplementary material for: Efficacy of foliar application of Chlorella vulgaris extract on chemical composition and biological activities of the essential oil of spearmint (Mentha spicata L.)
Source: Heliyon. 2024 Nov 22;10(23):e40531. doi: 10.1016/j.heliyon.2024.e40531 (PMC11629182; doi:10.1016/j.heliyon.2024.e40531)
Supplement: Multimedia component 1 [file mmc1.docx]

| **Data Filename** | F JK205 F S1.D | **Sample Name** | F JK205 F S1 |
| --- | --- | --- | --- |
| **Sample Type** |  | **Position** | 53 |
| **Instrument Name** | GCMSMS | **User Name** | DATASYSTEM01\admin |
| **Acq Method** | Essential Oil.M | **Acquired Time** | 4/27/2023 7:25:55 PM |
| **IRM Calibration Status** | Not Applicable | **DA Method** | Default1.m |
| **Comment** | Sample |  |  |
| **ExpectedBarcode315 78** | **SampleAmount3157 8** |  |  |
| **EquilibrationTime315 78** |  |  |  |
| **Data Filename** | F JK205 S L.D | **Sample Name** | F JK205 S L |
| **Sample Type** |  | **Position** | 52 |
| **Instrument Name** | GCMSMS | **User Name** | DATASYSTEM01\admin |
| **Acq Method** | Essential Oil.M | **Acquired Time** | 4/27/2023 6:18:12 PM |
| **IRM Calibration Status** | Not Applicable | **DA Method** | Default1.m |
| **Comment** | Sample |  |  |
| **ExpectedBarcode183 04** | **SampleAmount1830 4** |  |  |
| **EquilibrationTime183 04** |  |  |  |
| **Data Filename** | F JK205 S P 1 1.D | **Sample Name** | F JK205 S P 1 1 |
| **Sample Type** |  | **Position** | 41 |
| **Instrument Name** | GCMSMS | **User Name** | DATASYSTEM01\admin |
| **Acq Method** | Essential Oil.M | **Acquired Time** | 4/27/2023 5:54:20 AM |
| **IRM Calibration Status** | Not Applicable | **DA Method** | Default1.m |
| **Comment** | Sample |  |  |

# ExpectedBarcode344 0

**EquilibrationTime344 0**

# SampleAmount3440

| **Data Filename** | F JK205 S P 2 2.D | **Sample Name** | F JK205 S P 2 2 |
| --- | --- | --- | --- |
| **Sample Type** |  | **Position** | 42 |
| **Instrument Name** | GCMSMS | **User Name** | DATASYSTEM01\admin |
| **Acq Method** | Essential Oil.M | **Acquired Time** | 4/27/2023 7:01:06 AM |
| **IRM Calibration Status** | Not Applicable | **DA Method** | Default1.m |
| **Comment** | Sample |  |  |
| **ExpectedBarcode166 98** | **SampleAmount1669 8** |  |  |
| **EquilibrationTime166 98** |  |  |  |
| **Data Filename** | F JK205 S P 3 1.D | **Sample Name** | F JK205 S P 3 1 |
| **Sample Type** |  | **Position** | 43 |
| **Instrument Name** | GCMSMS | **User Name** | DATASYSTEM01\admin |
| **Acq Method** | Essential Oil.M | **Acquired Time** | 4/27/2023 8:08:47 AM |
| **IRM Calibration Status** | Not Applicable | **DA Method** | Default1.m |
| **Comment** | Sample |  |  |

**ExpectedBarcode299 63**

# EquilibrationTime299 63

**SampleAmount2996 3**

**User Chromatograms**

Fragmentor Voltage Collision Energy

0 Ionization Mode EI


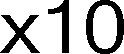

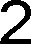


# Integration Peak List


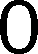

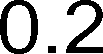

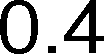

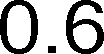

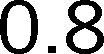

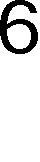

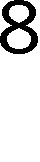

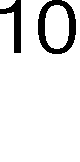

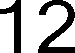

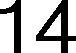

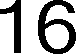

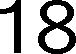

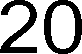

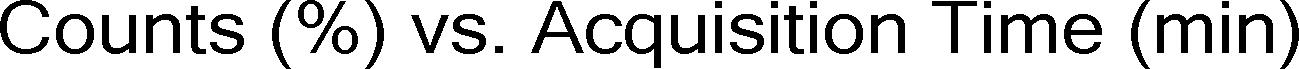

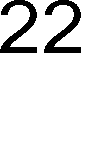

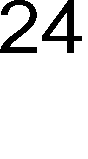

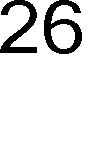

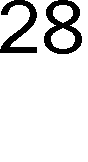

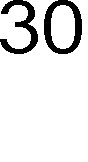

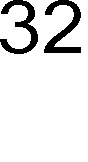

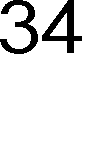

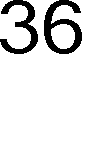

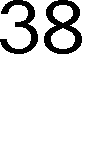

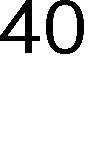

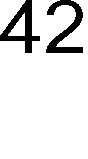

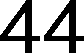

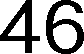

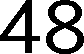

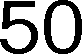

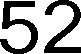

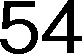

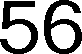

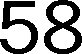

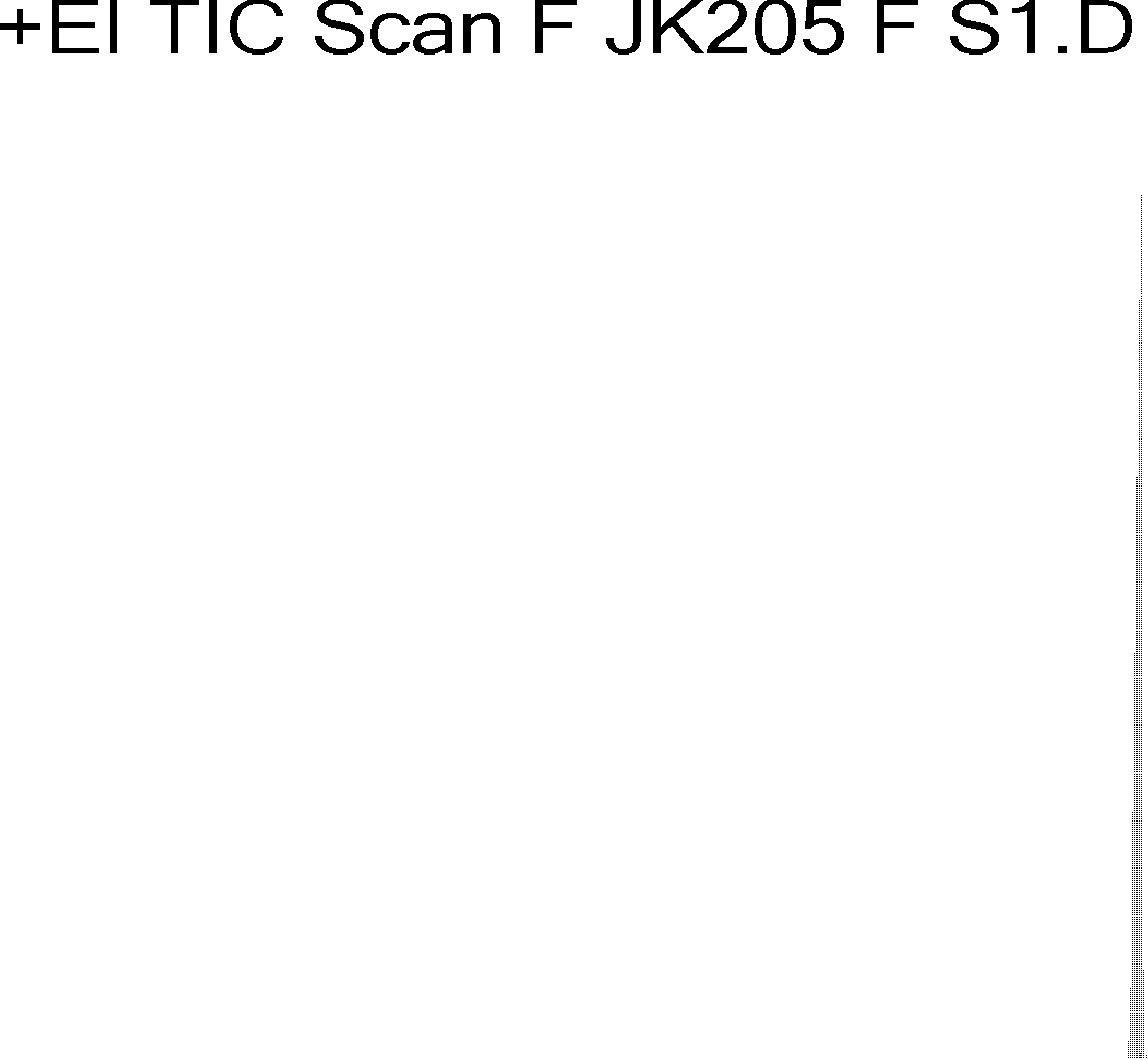

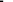

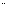

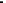

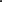

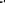

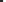

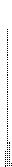

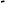

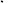

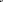

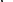

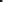

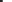

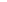

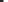

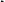

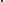

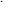

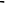

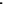

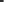

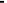

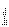

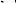

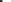

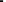

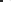

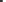

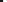

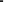

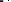

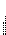

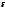

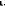

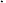

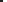

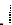

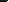

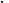

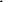

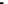

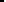

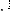

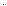

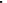

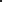

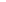

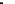

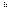

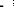

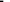

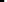

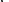

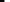

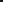

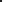

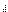

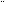

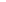


| **Peak** | **Start** | **RT** | **End** | **Height** | **Area** | **Area %** |  |
| --- | --- | --- | --- | --- | --- | --- | --- |
| **11** | **14**.**658** | **14**.**741** | **14**.**816** | **360650** | **998063** | 6.98 |  |
| 26 | 20.466 | 20.566 | 20.674 | 19961 | 75754 | 0.53 |  |
| 29 | 21.157 | 21.406 | 21.506 | 2159101 | 14299372 | 100 |  |
| 30 | 21.548 | 21.598 | 21.664 | 54424 | 151942 | 1.06 |  |
| 39 | 25.151 | 25.201 | 25.234 | 75215 | 221572 | 1.55 |  |
| 40 | 25.234 | 25.267 | 25.326 | 43781 | 122716 | 0.86 |  |
| 44 | 26.016 | 26.141 | 26.274 | 170895 | 529752 | 3.7 |  |
| 51 | 27.506 | 27.647 | 27.764 | 69227 | 210775 | 1.47 |  |
| 59 | 31.259 | 31.359 | 31.433 | 41939 | 133614 | 0.93 |  |
| 66 | 35.203 | 35.286 | 35.411 | 24248 | 63094 | 0.44 |  |

Fragmentor Voltage Collision Energy 0 Ionization Mode EI

# Integration Peak List

| **Peak** | **Start** | **RT** | **End** | **Height** | **Area** | **Area %** |
| --- | --- | --- | --- | --- | --- | --- |
| 11 | 14.666 | 14.733 | 14.799 | 89681 | 250866 | 5.23 |
| 24 | 19.85 | 19.9 | 20 | 24316 | 75178 | 1.57 |
| 29 | 21.157 | 21.323 | 21.506 | 1142477 | 4794122 | 100 |

| **Peak** | **Start** | **RT** | **End** | **Height** | **Area** | **Area %** |  |
| --- | --- | --- | --- | --- | --- | --- | --- |
| 34 | 23.345 | 23.412 | 23.537 | 17516 | 50573 | 1.05 |  |
| 37 | 25.151 | 25.201 | 25.234 | 53102 | 154268 | 3.22 |  |
| 38 | 25.234 | 25.259 | 25.317 | 19680 | 56879 | 1.19 |  |
| 42 | 26.025 | 26.141 | 26.216 | 109770 | 349217 | 7.28 |  |
| 49 | 27.589 | 27.647 | 27.731 | 28896 | 85985 | 1.79 |  |
| 54 | 30.127 | 30.177 | 30.252 | 29825 | 92095 | 1.92 |  |
| 56 | 31.267 | 31.367 | 31.433 | 25118 | 82250 | 1.72 |  |

Fragmentor Voltage Collision Energy 0 Ionization Mode EI

# Integration Peak List

| **Peak** | **Start** | **RT** | **End** | **Height** | **Area** | **Area %** |  |
| --- | --- | --- | --- | --- | --- | --- | --- |
| 11 | 14.684 | 14.767 | 14.825 | 696130 | 1972394 | 16.47 |  |
| 12 | 14.825 | 14.867 | 14.933 | 53990 | 140889 | 1.18 |  |
| 24 | 19.876 | 19.918 | 20.001 | 43853 | 129600 | 1.08 |  |
| 29 | 21.183 | 21.407 | 21.524 | 2019329 | 11972348 | 100 |  |
| 37 | 25.168 | 25.218 | 25.26 | 107007 | 313230 | 2.62 |  |
| 38 | 25.26 | 25.285 | 25.343 | 34534 | 88189 | 0.74 |  |
| 42 | 26.034 | 26.159 | 26.292 | 200908 | 642011 | 5.36 |  |
| 49 | 27.598 | 27.665 | 27.773 | 48850 | 144639 | 1.21 |  |
| 54 | 30.136 | 30.203 | 30.278 | 42647 | 130651 | 1.09 |  |
| 56 | 31.285 | 31.384 | 31.451 | 30057 | 96163 | 0.8 |  |

Fragmentor Voltage Collision Energy 0 Ionization Mode EI

# Integration Peak List

| **Peak** | **Start** | **RT** | **End** | **Height** | **Area** | **Area %** |
| --- | --- | --- | --- | --- | --- | --- |
| 11 | 14.675 | 14.766 | 14.824 | 539556 | 1521332 | 16.41 |

| **Peak** | **Start** | **RT** | **End** | **Height** | **Area** | **Area %** |  |
| --- | --- | --- | --- | --- | --- | --- | --- |
| 12 | 14.824 | 14.866 | 14.957 | 32032 | 82001 | 0.88 |  |
| 29 | 21.173 | 21.381 | 21.506 | 1739157 | 9269427 | 100 |  |
| 30 | 21.548 | 21.598 | 21.689 | 33543 | 96891 | 1.05 |  |
| 38 | 25.159 | 25.218 | 25.251 | 101181 | 294570 | 3.18 |  |
| 39 | 25.251 | 25.276 | 25.359 | 31674 | 88998 | 0.96 |  |
| 43 | 26.025 | 26.158 | 26.291 | 196189 | 619421 | 6.68 |  |
| 50 | 27.597 | 27.664 | 27.764 | 48739 | 142950 | 1.54 |  |
| 55 | 30.144 | 30.194 | 30.269 | 36389 | 113263 | 1.22 |  |
| 57 | 31.292 | 31.384 | 31.467 | 31863 | 97818 | 1.06 |  |

Fragmentor Voltage Collision Energy 0 Ionization Mode EI

# Integration Peak List

| **Peak** | **Start** | **RT** | **End** | **Height** | **Area** | **Area %** |
| --- | --- | --- | --- | --- | --- | --- |
| 11 | 14.675 | 14.749 | 14.816 | 180648 | 503035 | 12.87 |
| 12 | 14.824 | 14.866 | 14.966 | 10900 | 26655 | 0.68 |
| 29 | 21.173 | 21.323 | 21.481 | 998369 | 3909066 | 100 |
| 34 | 23.354 | 23.42 | 23.503 | 9985 | 27295 | 0.7 |
| 37 | 25.159 | 25.218 | 25.251 | 52176 | 158993 | 4.07 |
| 38 | 25.251 | 25.276 | 25.342 | 17641 | 49276 | 1.26 |
| 42 | 26.033 | 26.15 | 26.224 | 93341 | 303117 | 7.75 |
| 49 | 27.597 | 27.664 | 27.772 | 22574 | 68938 | 1.76 |
| 54 | 30.152 | 30.194 | 30.26 | 20077 | 59447 | 1.52 |
| 56 | 31.334 | 31.384 | 31.45 | 17419 | 49649 | 1.27 |

**User Spectra**

Spectrum Source

Peak (11) in "+ TIC Scan"

Collision Energy

0

Ionization Mode

EI

# Peak List

| **m/z** | **z** | **Abund** |
| --- | --- | --- |
| 53.1 |  | 7843 |
| 67.1 |  | 25490 |
| 68.1 | 1 | 33668 |
| 77 |  | 6731 |
| 79 |  | 11487 |
| 91 |  | 6854 |
| 92.1 |  | 6754 |
| 93.1 |  | 21035 |
| 94.1 |  | 7672 |
| 121.1 |  | 6268 |

Spectrum Source

Peak (29) in "+ TIC Scan"

Collision Energy

0

Ionization Mode

EI

**Peak List**

| **m/z** | **z** | **Abund** |
| --- | --- | --- |
| 41.1 |  | 29757 |
| 53.1 |  | 39120 |
| 54.1 |  | 107516 |
| 79.1 |  | 38843 |
| 82 | 1 | 299008 |
| 91 |  | 34375 |
| 93.1 | 1 | 93187 |
| 106.1 |  | 40178 |
| 107.1 |  | 55437 |
| 108.1 |  | 92870 |

Spectrum Source

Peak (30) in "+ TIC Scan"

Collision Energy

0

Ionization Mode

EI

**Peak List**

| **m/z** | **z** | **Abund** |
| --- | --- | --- |
| 41.1 |  | 1280 |
| 53 |  | 863 |
| 54 |  | 1125 |
| 55 |  | 912 |
| 82 | 1 | 6959 |
| 95 |  | 2462 |
| 109 |  | 1566 |
| 110.1 | 1 | 5144 |
| 137.1 |  | 1394 |
| 152. |  | 964 |

Spectrum Source

+ TIC Scan

Collision Energy

0

Ionization Mode

EI

**Peak List**

| **m/z** | **z** | **Abund** |
| --- | --- | --- |
| 41.1 |  | 1085 |
| 77 |  | 1472 |
| 79.1 |  | 3259 |
| 80.1 |  | 6966 |
| 81.1 | 1 | 9022 |
| 91 |  | 1314 |
| 105 |  | 964 |
| 123.1 | 1 | 5662 |
| 124.1 | 1 | 754 |
| 161.1 |  | 2223 |

Spectrum Source

Peak (44) in "+ TIC Scan"

Collision Energy

0

Ionization Mode

EI

**Peak List**

| **m/z** | **z** | **Abund** |
| --- | --- | --- |
| 41.1 |  | 5126 |
| 67.1 | 1 | 3226 |
| 69.1 | 1 | 6199 |
| 77 |  | 3408 |
| 79.1 |  | 5616 |
| 91 |  | 5753 |
| 93.1 | 1 | 6769 |
| 105 |  | 3459 |
| 107.1 | 1 | 2832 |
| 133.1 | 1 | 4965 |

Spectrum Source

Peak (51) in "+ TIC Scan"

Collision Energy

0

Ionization Mode

EI

**Peak List**

| **m/z** | **z** | **Abund** |
| --- | --- | --- |
| 41.1 |  | 1376 |
| 77 |  | 1624 |
| 79 |  | 1893 |
| 81.1 | 1 | 1912 |
| 91 | 1 | 2801 |
| 93.1 | 1 | 1296 |
| 105 | 1 | 2715 |
| 119.1 |  | 1548 |
| 120.1 | 1 | 1132 |
| 161.1 | 1 | 4273 |

Collision Energy

0

Ionization Mode

EI

**Peak List**

| **m/z** | **Abund** |
| --- | --- |
| 41 | 246 |
| 42.1 | 139 |
| 55 | 227 |
| 56.9 | 150 |
| 69 | 209 |
| 83 | 151 |

Collision Energy

0

Ionization Mode

EI

**Peak List**

| **m/z** | **Abund** |
| --- | --- |
| 41 | 124 |
| 42.1 | 109 |
| 43.1 | 125 |
| 55 | 344 |
| 57 | 158 |
| 70 | 110 |
| 81 | 445 |
| 99.1 | 295 |

Collision Energy

0

Ionization Mode

EI

**Peak List**

| **m/z** | **z** | **Abund** |
| --- | --- | --- |
| 41 |  | 372 |
| 53 |  | 242 |
| 77 |  | 791 |
| 79 |  | 690 |
| 91 |  | 1085 |
| 92 |  | 917 |
| 93.1 | 1 | 2553 |
| 94 | 1 | 255 |
| 105 |  | 257 |
| 121. |  | 315 |

Collision Energy

0

Ionization Mode

EI

**Peak List**

| **m/z** | **z** | **Abund** |
| --- | --- | --- |
| 41.1 |  | 296 |
| 55 |  | 187 |
| 68.9 |  | 189 |
| 77 |  | 620 |
| 79.1 |  | 459 |
| 80 |  | 177 |
| 91 | 1 | 680 |
| 93.1 | 1 | 1455 |
| 94.1 | 1 | 202 |
| 136.1 |  | 221 |

Collision Energy

0

Ionization Mode

EI

**Peak List**

| **m/z** | **z** | **Abund** |
| --- | --- | --- |
| 41.1 |  | 1018 |
| 53 |  | 405 |
| 67 |  | 424 |
| 69.1 |  | 1143 |
| 77 | 1 | 849 |
| 79 |  | 922 |
| 91 |  | 923 |
| 92 |  | 401 |
| 93.1 | 1 | 3299 |
| 94 | 1 | 440 |

Collision Energy

0

Ionization Mode

EI

**Peak List**

| **m/z** | **z** | **Abund** |
| --- | --- | --- |
| 41.1 |  | 1231 |
| 53 |  | 268 |
| 67.1 |  | 271 |
| 69.1 |  | 1415 |
| 77 |  | 338 |
| 78.9 |  | 285 |
| 91 |  | 393 |
| 92 |  | 210 |
| 93 | 1 | 1672 |
| 94 | 1 | 183 |

Collision Energy

0

Ionization Mode

EI

**Peak List**

| **m/z** | **z** | **Abund** |
| --- | --- | --- |
| 41.1 |  | 905 |
| 43.1 |  | 540 |
| 44.1 |  | 288 |
| 55 | 1 | 2215 |
| 56.1 | 1 | 260 |
| 57 |  | 564 |
| 58 |  | 393 |
| 59.1 |  | 4187 |
| 83.1 |  | 2148 |
| 101.1 |  | 900 |

Collision Energy

0

Ionization Mode

EI

**Peak List**

| **m/z** | **Abund** |
| --- | --- |
| 41.1 | 134 |
| 43 | 136 |
| 43.9 | 124 |
| 55.1 | 116 |
| 56 | 104 |
| 57 | 168 |

Collision Energy

0

Ionization Mode

EI

**Peak List**

| **m/z** | **Abund** |
| --- | --- |
| 93.1 | 108 |

Collision Energy

0

Ionization Mode

EI

**Peak List**

| **m/z** | **z** | **Abund** |
| --- | --- | --- |
| 41 |  | 384 |
| 43 |  | 752 |
| 55 |  | 353 |
| 67 |  | 503 |
| 68.1 |  | 519 |
| 71 |  | 364 |
| 78.9 |  | 419 |
| 81 |  | 559 |
| 84 |  | 359 |
| 93 | 1 | 898 |

Collision Energy

0

Ionization Mode

EI

**Peak List**

| **m/z** | **Abund** |
| --- | --- |
| 41.1 | 105 |
| 64.9 | 131 |
| 77 | 169 |
| 78.9 | 194 |
| 91 | 440 |
| 92 | 175 |
| 93 | 350 |

Collision Energy

0

Ionization Mode

EI

**Peak List**

| **m/z** | **Abund** |
| --- | --- |
| 41 | 191 |
| 43 | 284 |
| 55 | 242 |
| 67.1 | 112 |
| 69 | 166 |
| 71.1 | 396 |
| 80 | 116 |
| 93 | 226 |

Collision Energy

0

Ionization Mode

EI

**Peak List**

| **m/z** | **Abund** |
| --- | --- |
| 41 | 111 |
| 43.2 | 163 |
| 55 | 169 |
| 57 | 293 |
| 70.1 | 355 |
| 85 | 145 |

Collision Energy

0

Ionization Mode

EI

**Peak List**

| **m/z** | **Abund** |
| --- | --- |
| 41.1 | 503 |
| 43.1 | 504 |
| 44 | 348 |
| 55 | 444 |
| 56.1 | 378 |
| 57.1 | 734 |
| 68.1 | 210 |
| 69 | 268 |
| 70 | 343 |
| 81 | 201 |

Collision Energy

0

Ionization Mode

EI

**Peak List**

| **m/z** | **Abund** |
| --- | --- |
| 41 | 131 |
| 43 | 1086 |
| 54.9 | 155 |
| 56.1 | 102 |
| 57.1 | 116 |
| 70.1 | 130 |
| 83.1 | 130 |
| 101 | 203 |

Collision Energy

0

Ionization Mode

EI

**Peak List**

| **m/z** | **Abund** |
| --- | --- |
| 41.1 | 167 |
| 42.9 | 301 |
| 67 | 137 |
| 79 | 315 |
| 81 | 157 |
| 91 | 165 |
| 94 | 281 |
| 94.9 | 147 |
| 109 | 282 |
| 137 | 157 |

Collision Energy

0

Ionization Mode

EI

**Peak List**

| **m/z** | **Abund** |
| --- | --- |
| 41 | 220 |
| 43.1 | 356 |
| 55.1 | 139 |
| 57 | 136 |
| 67 | 297 |
| 68.9 | 135 |
| 78.9 | 180 |
| 81 | 178 |
| 94 | 204 |
| 108 | 161 |

Collision Energy

0

Ionization Mode

EI

**Peak List**

| **m/z** | **Abund** |
| --- | --- |
| 41 | 176 |
| 43.1 | 186 |
| 55 | 142 |
| 67.1 | 145 |
| 69 | 248 |
| 71 | 404 |
| 76.9 | 101 |
| 93 | 170 |
| 111.1 | 166 |

Collision Energy

0

Ionization Mode

EI

**Peak List**

| **m/z** | **Abund** |
| --- | --- |
| 41.1 | 159 |
| 55 | 167 |
| 59 | 104 |
| 66.9 | 117 |
| 69 | 198 |
| 90.9 | 136 |
| 109 | 187 |
| 119 | 113 |
| 134 | 120 |

Collision Energy

0

Ionization Mode

EI

**Peak List**

| **m/z** | **Abund** |
| --- | --- |
| 41.1 | 142 |
| 43.1 | 266 |
| 59 | 791 |
| 67 | 201 |
| 79 | 146 |
| 81 | 287 |
| 92 | 150 |
| 93 | 412 |
| 121 | 378 |
| 136 | 250 |

Collision Energy

0

Ionization Mode

EI

**Peak List**

| **m/z** | **z** | **Abund** |
| --- | --- | --- |
| 41 |  | 830 |
| 53 |  | 553 |
| 55.1 |  | 709 |
| 67 |  | 2107 |
| 68.1 |  | 1016 |
| 69.1 | 1 | 752 |
| 81 |  | 850 |
| 82 | 1 | 852 |
| 95.1 | 1 | 1343 |
| 109 |  | 636 |

Collision Energy

0

Ionization Mode

EI

**Peak List**

| **m/z** | **Abund** |
| --- | --- |
| 41.1 | 239 |
| 43 | 187 |
| 55.1 | 228 |
| 66.9 | 249 |
| 79 | 318 |
| 81 | 199 |
| 93 | 317 |
| 107 | 285 |
| 121 | 282 |

| **m/z** | **Abund** |
| --- | --- |
| 136.1 | 220 |

Collision Energy

0

Ionization Mode

EI

**Peak List**

| **m/z** | **Abund** |
| --- | --- |
| 41 | 107 |
| 54.9 | 114 |
| 67.1 | 209 |
| 68.1 | 110 |
| 95 | 128 |

Collision Energy

0

Ionization Mode

EI

**Peak List**

| **m/z** | **z** | **Abund** |
| --- | --- | --- |
| 41.1 |  | 824 |
| 55 |  | 1038 |
| 67 |  | 621 |
| 69 |  | 787 |
| 77 |  | 544 |
| 83 |  | 987 |
| 84 | 1 | 1776 |
| 91 |  | 702 |
| 95 |  | 542 |
| 109 | 1 | 2459 |

Collision Energy

0

Ionization Mode

EI

**Peak List**

| **m/z** | **z** | **Abund** |
| --- | --- | --- |
| 41.1 |  | 408 |
| 41.9 |  | 116 |
| 43 |  | 188 |
| 54.9 |  | 306 |
| 57.1 |  | 636 |
| 67 |  | 915 |
| 69.2 |  | 122 |
| 82.1 | 1 | 999 |
| 83 | 1 | 153 |
| 85 |  | 575 |

Collision Energy

0

Ionization Mode

EI

**Peak List**

| **m/z** | **z** | **Abund** |
| --- | --- | --- |
| 41.1 |  | 529 |
| 43.1 |  | 372 |
| 55 |  | 660 |
| 67 |  | 375 |
| 69 |  | 534 |
| 83 |  | 609 |
| 84 | 1 | 1244 |
| 91 |  | 409 |
| 109 | 1 | 621 |
| 134.1 |  | 497 |

Collision Energy

0

Ionization Mode

EI

**Peak List**

| **m/z** | **Abund** |
| --- | --- |
| 41 | 639 |
| 43 | 2202 |
| 53 | 682 |
| 55 | 737 |
| 67.1 | 1594 |
| 68 | 713 |
| 79 | 613 |
| 85 | 1298 |
| 95 | 869 |
| 123 | 870 |

Collision Energy

0

Ionization Mode

EI

**Peak List**

| **m/z** | **Abund** |
| --- | --- |
| 41 | 232 |
| 43 | 301 |
| 55 | 229 |
| 69 | 538 |
| 84.1 | 265 |
| 90.9 | 212 |
| 95 | 196 |
| 107 | 373 |

| **m/z** | **Abund** |
| --- | --- |
| 108.9 | 132 |
| 179.1 | 649 |

Collision Energy

0

Ionization Mode

EI

**Peak List**

| **m/z** | **z** | **Abund** |
| --- | --- | --- |
| 41.1 |  | 293 |
| 43 |  | 306 |
| 55 |  | 267 |
| 69 |  | 527 |
| 77 |  | 186 |
| 79 |  | 171 |
| 91 |  | 238 |
| 95.1 |  | 173 |
| 106.9 |  | 265 |
| 179.1 | 1 | 1207 |

Collision Energy

0

Ionization Mode

EI

**Peak List**

| **m/z** | **Abund** |
| --- | --- |
| 41 | 108 |
| 55 | 393 |
| 67.1 | 410 |
| 82 | 449 |
| 83 | 432 |

Collision Energy

0

Ionization Mode

EI

**Peak List**

| **m/z** | **Abund** |
| --- | --- |
| 41.1 | 163 |
| 43 | 464 |
| 67 | 153 |
| 67.9 | 128 |
| 78.9 | 195 |
| 93 | 310 |
| 94.1 | 136 |
| 107 | 280 |
| 121 | 217 |
| 136 | 168 |

Collision Energy

0

Ionization Mode

EI

**Peak List**

| **m/z** | **Abund** |
| --- | --- |
| 43.1 | 223 |
| 84.1 | 165 |
| 90.9 | 132 |
| 109 | 180 |
| 119 | 138 |
| 151.9 | 119 |

Collision Energy

0

Ionization Mode

EI

**Peak List**

| **m/z** | **z** | **Abund** |
| --- | --- | --- |
| 41 |  | 205 |
| 53 |  | 199 |
| 77 |  | 217 |
| 79 |  | 317 |
| 81.9 |  | 260 |
| 91 |  | 331 |
| 107 | 1 | 709 |
| 109 | 1 | 247 |
| 135 |  | 295 |
| 150. |  | 904 |

Collision Energy

0

Ionization Mode

EI

**Peak List**

| **m/z** | **Abund** |
| --- | --- |
| 43.1 | 393 |
| 55 | 273 |
| 76.9 | 360 |
| 79 | 187 |
| 84 | 391 |
| 91 | 408 |
| 103 | 230 |
| 109 | 282 |
| 119 | 323 |
| 164.1 | 483 |

Collision Energy

0

Ionization Mode

EI

**Peak List**

| **m/z** | **z** | **Abund** |
| --- | --- | --- |
| 41.1 | 1 | 1731 |
| 53 |  | 1720 |
| 67 |  | 2900 |
| 68 |  | 2592 |
| 77 | 1 | 1469 |
| 79 |  | 2541 |
| 81.1 | 1 | 4240 |
| 91.1 |  | 1724 |
| 93.1 |  | 3320 |
| 107.1 |  | 1859 |

Collision Energy

0

Ionization Mode

EI

**Peak List**

| **m/z** | **z** | **Abund** |
| --- | --- | --- |
| 55 |  | 803 |
| 77 |  | 822 |
| 79 | 1 | 1478 |
| 91 | 1 | 963 |
| 93 | 1 | 695 |
| 110 | 1 | 929 |
| 122 | 1 | 641 |
| 135 | 1 | 637 |
| 149 |  | 723 |
| 164.1 | 1 | 916 |

Collision Energy

0

Ionization Mode

EI

**Peak List**

| **m/z** | **Abund** |
| --- | --- |
| 41 | 109 |
| 43 | 109 |
| 71 | 118 |
| 79.1 | 111 |
| 90.9 | 175 |
| 93 | 117 |
| 105. | 118 |
| 107 | 195 |
| 119 | 131 |
| 121 | 148 |

Collision Energy

0

Ionization Mode

EI

**Peak List**

| **m/z** | **Abund** |
| --- | --- |
| 41 | 339 |
| 55.1 | 170 |
| 67.1 | 168 |
| 68.9 | 247 |
| 77 | 193 |
| 79 | 319 |
| 91 | 280 |
| 93 | 352 |

| **m/z** | **Abund** |
| --- | --- |
| 104.9 | 177 |
| 133 | 172 |

Collision Energy

0

Ionization Mode

EI

**Peak List**

| **m/z** | **z** | **Abund** |
| --- | --- | --- |
| 41 |  | 305 |
| 77 |  | 364 |
| 79 |  | 385 |
| 81 |  | 329 |
| 91 | 1 | 658 |
| 93 | 1 | 355 |
| 105 |  | 729 |
| 119 |  | 363 |
| 120.1 |  | 294 |
| 161.1 | 1 | 1411 |

Collision Energy

0

Ionization Mode

EI

**Peak List**

| **m/z** | **z** | **Abund** |
| --- | --- | --- |
| 41 |  | 932 |
| 43.1 |  | 390 |
| 55.1 |  | 396 |
| 57.1 |  | 481 |
| 67 |  | 504 |
| 69.1 | 1 | 1765 |

| **m/z** | **z** | **Abund** |
| --- | --- | --- |
| 71 |  | 405 |
| 79 |  | 388 |
| 81 |  | 341 |
| 93 | 1 | 875 |

Collision Energy

0

Ionization Mode

EI

**Peak List**

| **m/z** | **z** | **Abund** |
| --- | --- | --- |
| 41.1 |  | 519 |
| 55 |  | 356 |
| 77 |  | 569 |
| 79 |  | 625 |
| 81 |  | 648 |
| 91.1 | 1 | 920 |
| 93 | 1 | 576 |
| 105 | 1 | 1024 |
| 119 |  | 568 |
| 161.2 | 1 | 1563 |

Collision Energy

0

Ionization Mode

EI

**Peak List**

| **m/z** | **z** | **Abund** |
| --- | --- | --- |
| 41.1 |  | 310 |
| 67 |  | 294 |
| 77 |  | 301 |
| 79 |  | 338 |

| **m/z** | **z** | **Abund** |
| --- | --- | --- |
| 80 |  | 531 |
| 91 |  | 321 |
| 92 |  | 283 |
| 93.1 | 1 | 1499 |
| 121.1 | 1 | 370 |
| 147.1 |  | 236 |

Collision Energy

0

Ionization Mode

EI

**Peak List**

| **m/z** | **z** | **Abund** |
| --- | --- | --- |
| 41.1 |  | 594 |
| 76.9 |  | 604 |
| 79 |  | 585 |
| 81.1 |  | 602 |
| 91 |  | 1415 |
| 105.1 |  | 1406 |
| 119 |  | 861 |
| 161.1 | 1 | 4470 |
| 162.1 | 1 | 660 |
| 204.2 |  | 738 |

Collision Energy

0

Ionization Mode

EI

**Peak List**

| **m/z** | **Abund** |
| --- | --- |
| 40.9 | 238 |
| 55 | 172 |

| **m/z** | **Abund** |
| --- | --- |
| 77 | 237 |
| 79 | 279 |
| 81 | 228 |
| 91 | 382 |
| 93 | 214 |
| 105 | 334 |
| 119 | 206 |
| 161.1 | 575 |

Collision Energy

0

**Peak List**

| **m/z** | **Abund** |
| --- | --- |
| 41 | 129 |
| 79 | 121 |
| 91 | 102 |
| 93.1 | 102 |
| 105 | 104 |
| 121 | 113 |

Collision Energy

0

Ionization Mode

EI

Ionization Mode

EI

**Peak List**

| **m/z** | **z** | **Abund** |
| --- | --- | --- |
| 41.1 |  | 326 |
| 76.9 |  | 341 |
| 79 |  | 455 |
| 81 |  | 280 |

| **m/z** | **z** | **Abund** |
| --- | --- | --- |
| 91 |  | 543 |
| 93 |  | 415 |
| 105 |  | 559 |
| 119 |  | 417 |
| 133.1 |  | 301 |
| 161.1 | 1 | 1226 |

Collision Energy

0

Ionization Mode

EI

**Peak List**

| **m/z** | **z** | **Abund** |
| --- | --- | --- |
| 105 |  | 271 |
| 115 |  | 345 |
| 128 |  | 553 |
| 129 |  | 590 |
| 131 |  | 371 |
| 143 |  | 250 |
| 144 |  | 378 |
| 159.1 | 1 | 4800 |
| 160.1 | 1 | 605 |
| 202.1 |  | 496 |

Collision Energy

0

Ionization Mode

EI

**Peak List**

| **m/z** | **Abund** |
| --- | --- |
| 40.9 | 119 |
| 42.9 | 113 |

| **m/z** | **Abund** |
| --- | --- |
| 54.9 | 121 |
| 66.9 | 100 |
| 109 | 116 |
| 137.1 | 127 |
| 207.2 | 130 |

Collision Energy

0

Ionization Mode

EI

**Peak List**

| **m/z** | **Abund** |
| --- | --- |
| 41 | 139 |
| 43.1 | 118 |
| 55 | 122 |
| 77 | 126 |
| 78.9 | 132 |
| 80.9 | 182 |
| 91 | 196 |
| 93 | 157 |
| 105 | 526 |
| 161.1 | 248 |

Collision Energy

0

Ionization Mode

EI

**Peak List**

| **m/z** | **z** | **Abund** |
| --- | --- | --- |
| 41.1 | 1 | 1037 |
| 43.1 | 1 | 1239 |
| 55 |  | 713 |

| **m/z** | **z** | **Abund** |
| --- | --- | --- |
| 67 |  | 663 |
| 69 |  | 862 |
| 76.9 |  | 621 |
| 79 | 1 | 1367 |
| 91 | 1 | 967 |
| 93 | 1 | 1045 |
| 95 |  | 704 |

Collision Energy

0

Ionization Mode

EI

**Peak List**

| **m/z** | **z** | **Abund** |
| --- | --- | --- |
| 41 |  | 551 |
| 43.1 |  | 509 |
| 55 | 1 | 570 |
| 81.1 |  | 477 |
| 95 |  | 552 |
| 105 |  | 595 |
| 119 | 1 | 1065 |
| 161.1 | 1 | 1054 |
| 179 | 1 | 728 |
| 204.1 |  | 453 |

Collision Energy

0

Ionization Mode

EI

**Peak List**

| **m/z** | **z** | **Abund** |
| --- | --- | --- |
| 41.1 | 13 | 10 |

| **m/z** | **z** | **Abund** |
| --- | --- | --- |
| 43.1 |  | 2451 |
| 79 |  | 1614 |
| 81.1 |  | 1775 |
| 91 | 1 | 1333 |
| 93 | 1 | 1188 |
| 95.1 |  | 1375 |
| 105 | 1 | 1812 |
| 161.1 | 1 | 5788 |
| 204.2 | 1 | 2211 |

Collision Energy

0

**Peak List**

Collision Energy

| **m/z** | **z** | **Abund** |
| --- | --- | --- |
| 41.1 |  | 382 |
| 43 |  | 829 |
| 55 |  | 372 |
| 71 |  | 417 |
| 79 |  | 521 |
| 81 |  | 481 |
| 95 | 1 | 1148 |
| 109.1 |  | 388 |
| 121.1 | 1 | 837 |
| 161 |  | 392 |

0

Ionization Mode

EI

Ionization Mode

EI

# Peak List

| **m/z** | **Abund** |
| --- | --- |
| 41 | 252 |
| 43 | 196 |
| 55 | 239 |
| 67 | 221 |
| 69 | 159 |
| 78.9 | 176 |
| 81 | 165 |
| 90.9 | 160 |
| 93 | 171 |
| 94.9 | 150 |

Collision Energy

0

**Peak List**

| **m/z** | **Abund** |
| --- | --- |
| 41 | 161 |
| 43 | 130 |
| 55 | 176 |
| 66.9 | 110 |
| 77.1 | 113 |
| 78.9 | 173 |
| 90.9 | 170 |
| 92.9 | 156 |
| 94.9 | 114 |

Collision Energy

0

Ionization Mode

EI

Ionization Mode

EI

**Peak List**

| **m/z** | **Abund** |
| --- | --- |
| 41 | 191 |
| 43 | 183 |
| 55.1 | 191 |
| 67 | 192 |
| 76.9 | 162 |
| 78.9 | 204 |
| 81 | 190 |
| 90.9 | 255 |
| 92.9 | 153 |
| 109. | 242 |

Collision Energy

0

Ionization Mode

EI

**Peak List**

| **m/z** | **Abund** |
| --- | --- |
| 41.1 | 1005 |
| 43.1 | 3594 |
| 55 | 1385 |
| 57.1 | 1357 |
| 58 | 3187 |
| 59.1 | 1470 |
| 69.1 | 972 |
| 71 | 1823 |
| 85.1 | 902 |
| 95 | 654 |

Collision Energy

0

Ionization Mode

EI

**Peak List**

| **m/z** | **Abund** |
| --- | --- |
| 41 | 138 |
| 50.9 | 111 |
| 55.1 | 101 |
| 67 | 541 |
| 77 | 481 |
| 82.1 | 608 |
| 104.9 | 734 |

Collision Energy

0

Ionization Mode

EI

**Peak List**

| **m/z** | **Abund** |
| --- | --- |
| 40.9 | 150 |
| 57 | 270 |
| 104 | 107 |
| 149 | 863 |

Collision Energy

0

Ionization Mode

EI

**Peak List**

| **m/z** | **Abund** |
| --- | --- |
| 41 | 213 |
| 43 | 317 |
| 66.9 | 134 |
| 69 | 446 |
| 81 | 152 |
| 95 | 100 |

Collision Energy

0

Ionization Mode

EI

**Peak List**

| **m/z** | **Abund** |
| --- | --- |
| 41 | 162 |
| 43 | 160 |
| 55 | 167 |
| 56.9 | 215 |
| 69.1 | 112 |
| 78.9 | 114 |
| 81 | 112 |
| 82 | 100 |
| 97 | 356 |
| 164.1 | 104 |

Collision Energy

0

Ionization Mode

EI

**Peak List**

| **m/z** | **Abund** |
| --- | --- |
| 41.1 | 102 |
| 92.9 | 126 |

Collision Energy

0

Ionization Mode

EI

**Peak List**

| **m/z** | **Abund** |
| --- | --- |
| 92.9 | 112 |

Spectrum Source

Peak (11) in "+ TIC Scan"

Collision Energy

0

Ionization Mode

EI

**Peak List**

| **m/z** | **z** | **Abund** |
| --- | --- | --- |
| 53 |  | 2209 |
| 67.1 |  | 7116 |

| **m/z** | **z** | **Abund** |
| --- | --- | --- |
| 68.1 | 1 | 9134 |
| 77 |  | 1832 |
| 79 |  | 3135 |
| 91 |  | 1902 |
| 92 |  | 1836 |
| 93 |  | 5606 |
| 94.1 |  | 2097 |
| 121.1 |  | 1718 |

Spectrum Source

+ TIC Scan

Collision Energy

0

Ionization Mode

EI

**Peak List**

| **m/z** | **z** | **Abund** |
| --- | --- | --- |
| 41 |  | 727 |
| 53 |  | 401 |
| 55 |  | 585 |
| 67.1 |  | 1975 |
| 68 |  | 1016 |
| 69 |  | 673 |
| 81 |  | 792 |
| 82 | 1 | 873 |
| 95 | 1 | 1298 |
| 109 |  | 632 |

Spectrum Source

Peak (29) in "+ TIC Scan"

Collision Energy

0

Ionization Mode

EI

# Peak List

| **m/z** | **z** | **Abund** |
| --- | --- | --- |
| 41.1 |  | 17875 |
| 53 |  | 23082 |
| 54.1 |  | 60247 |
| 79.1 |  | 21252 |
| 82 | 1 | 156504 |
| 91 |  | 18616 |
| 93.1 | 1 | 48178 |
| 106.1 |  | 20202 |
| 107.1 |  | 28530 |
| 108.1 |  | 46630 |

Spectrum Source

Peak (34) in "+ TIC Scan"

**Peak List**

| **m/z** | **z** | **Abund** |
| --- | --- | --- |
| 41 |  | 363 |
| 43.1 |  | 1287 |
| 67 |  | 327 |
| 68 |  | 333 |
| 79 |  | 510 |
| 93 |  | 909 |
| 107 | 1 | 435 |
| 107.1 |  | 369 |
| 121.1 |  | 727 |
| 136.1 |  | 577 |

Spectrum Source

+ TIC Scan

Collision Energy

0

Collision Energy

0

Ionization Mode

EI

Ionization Mode

EI

**Peak List**

| **m/z** | **z** | **Abund** |
| --- | --- | --- |
| 41.1 |  | 859 |
| 77 |  | 1063 |
| 79 |  | 2351 |
| 80 |  | 4721 |
| 81.1 | 1 | 6292 |
| 91 |  | 1009 |
| 105 |  | 728 |
| 123.1 | 1 | 3820 |
| 124.1 | 1 | 503 |
| 161. |  | 1537 |

Spectrum Source

Peak (42) in "+ TIC Scan"

Collision Energy

0

Ionization Mode

EI

**Peak List**

| **m/z** | **z** | **Abund** |
| --- | --- | --- |
| 41.1 | 1 | 3202 |
| 67 | 1 | 2013 |
| 69 | 1 | 3822 |
| 77 |  | 2158 |
| 79 |  | 3502 |
| 91 |  | 3497 |
| 93 | 1 | 4079 |
| 105 |  | 2113 |
| 107.1 | 1 | 1731 |
| 133.1 | 1 | 3006 |

Spectrum Source

Peak (49) in "+ TIC Scan"

Collision Energy

0

Ionization Mode

EI

**Peak List**

| **m/z** | **z** | **Abund** |
| --- | --- | --- |
| 41.1 |  | 637 |
| 77 |  | 759 |
| 79 | 1 | 848 |
| 81.1 |  | 473 |
| 91 | 1 | 1205 |
| 93 | 1 | 561 |
| 105 | 1 | 1173 |
| 119 |  | 682 |
| 120.1 | 1 | 491 |
| 161.1 | 1 | 1878 |

Spectrum Source

+ TIC Scan

Collision Energy

0

Ionization Mode

EI

**Peak List**

| **m/z** | **z** | **Abund** |
| --- | --- | --- |
| 41.1 |  | 944 |
| 43.1 |  | 1022 |
| 55 |  | 567 |
| 67 |  | 569 |
| 69.1 |  | 732 |
| 77 |  | 588 |
| 79 | 1 | 1178 |
| 91 | 1 | 840 |
| 93 | 1 | 794 |
| 109 |  | 483 |

Spectrum Source

+ TIC Scan

Collision Energy

0

Ionization Mode

EI

**Peak List**

| **m/z** | **z** | **Abund** |
| --- | --- | --- |
| 41.1 |  | 555 |
| 43 |  | 808 |
| 79 |  | 635 |
| 81.1 | 1 | 625 |
| 91 | 1 | 532 |
| 93.1 | 1 | 455 |
| 95 |  | 536 |
| 105 |  | 647 |
| 161.1 | 1 | 1910 |
| 204.1 | 1 | 721 |

Collision Energy

0

Ionization Mode

EI

Collision Energy

0

Ionization Mode

EI

**Peak List**

| **m/z** | **Abund** |
| --- | --- |
| 54.9 | 133 |
| 80.9 | 154 |
| 99 | 100 |

Collision Energy

0

Ionization Mode

EI

**Peak List**

| **m/z** | **Abund** |
| --- | --- |
| 41.1 | 124 |
| 76.9 | 215 |
| 78.9 | 233 |
| 91 | 316 |
| 92 | 272 |
| 93 | 758 |

Collision Energy

0

Ionization Mode

EI

**Peak List**

| **m/z** | **Abund** |
| --- | --- |
| 40.8 | 111 |
| 77 | 191 |
| 79 | 141 |
| 90.9 | 240 |
| 93 | 539 |

Collision Energy

0

**Peak List**

Collision Energy

| **m/z** | **z** | **Abund** |
| --- | --- | --- |
| 41.1 |  | 364 |
| 53 |  | 141 |
| 67 |  | 168 |
| 69.1 |  | 459 |
| 77 |  | 357 |
| 79.1 |  | 298 |
| 91 |  | 377 |
| 93 | 1 | 1171 |
| 94 | 1 | 152 |
| 121.1 |  | 157 |

0

Ionization Mode

EI

Ionization Mode

EI

**Peak List**

| **m/z** | **Abund** |
| --- | --- |
| 41 | 505 |
| 52.9 | 112 |
| 67 | 127 |
| 69 | 610 |
| 76.9 | 108 |
| 78.9 | 124 |
| 91 | 176 |
| 93 | 688 |

Collision Energy

0

Ionization Mode

EI

**Peak List**

| **m/z** | **z** | **Abund** |
| --- | --- | --- |
| 41.1 |  | 425 |
| 43 |  | 266 |
| 44.1 |  | 165 |
| 55 | 1 | 1013 |
| 56 | 1 | 132 |
| 57 |  | 279 |
| 58 |  | 168 |
| 59 |  | 1810 |
| 83.1 |  | 996 |
| 101.1 |  | 423 |

Collision Energy

0

Ionization Mode

EI

Collision Energy

0

Ionization Mode

EI

Collision Energy

0

Ionization Mode

EI

**Peak List**

| **m/z** | **Abund** |
| --- | --- |
| 41 | 479 |
| 43.1 | 1028 |
| 55 | 460 |
| 67.1 | 419 |
| 69.1 | 420 |
| 71 | 533 |
| 81.1 | 698 |
| 84 | 467 |

| **m/z** | **Abund** |
| --- | --- |
| 93 | 910 |
| 108 | 532 |

Collision Energy

0

Ionization Mode

EI

**Peak List**

| **m/z** | **Abund** |
| --- | --- |
| 41 | 127 |
| 77 | 142 |
| 79 | 177 |
| 80 | 145 |
| 90.9 | 198 |
| 93 | 322 |

Collision Energy

0

Ionization Mode

EI

**Peak List**

| **m/z** | **Abund** |
| --- | --- |
| 55.1 | 246 |
| 59 | 298 |
| 97 | 159 |

Collision Energy

0

Ionization Mode

EI

**Peak List**

| **m/z** | **z** | **Abund** |
| --- | --- | --- |
| 41 |  | 143 |
| 43 |  | 210 |
| 55.1 |  | 173 |
| 57.1 |  | 334 |
| 70 | 1 | 455 |
| 71.1 | 1 | 101 |
| 85 |  | 185 |

Collision Energy

0

Ionization Mode

EI

**Peak List**

| **m/z** | **Abund** |
| --- | --- |
| 41 | 201 |
| 43 | 202 |
| 44.1 | 124 |
| 55.2 | 134 |
| 56 | 131 |
| 57.1 | 284 |
| 70.1 | 170 |

Collision Energy

0

Ionization Mode

EI

**Peak List**

| **m/z** | **z** | **Abund** |
| --- | --- | --- |
| 41.1 |  | 185 |
| 43.1 | 1 | 1692 |
| 44.1 | 1 | 117 |
| 55.1 |  | 217 |
| 56.1 |  | 176 |
| 57 |  | 127 |
| 70 |  | 201 |
| 83.1 |  | 212 |
| 101 |  | 321 |
| 112. |  | 131 |

Collision Energy

0

Collision Energy

0

Ionization Mode

EI

Ionization Mode

EI

**Peak List**

| **m/z** | **Abund** |
| --- | --- |
| 41 | 141 |
| 43 | 118 |
| 56.9 | 135 |
| 67 | 102 |

Collision Energy

0

**Peak List**

| **m/z** | **Abund** |
| --- | --- |
| 41.2 | 119 |
| 43 | 169 |
| 55 | 138 |
| 67.1 | 124 |
| 69 | 135 |
| 71 | 366 |
| 93 | 197 |
| 111.1 | 152 |

Collision Energy

0

Ionization Mode

EI

Ionization Mode

EI

**Peak List**

| **m/z** | **Abund** |
| --- | --- |
| 58.9 | 104 |
| 69 | 118 |

Collision Energy

0

Ionization Mode

EI

**Peak List**

| **m/z** | **Abund** |
| --- | --- |
| 43.1 | 104 |
| 59.1 | 218 |
| 81 | 104 |
| 93 | 143 |
| 121 | 122 |

Collision Energy

0

Ionization Mode

EI

**Peak List**

| **m/z** | **z** | **Abund** |
| --- | --- | --- |
| 41.1 |  | 600 |
| 55.1 |  | 609 |
| 67.1 |  | 653 |
| 69 |  | 475 |
| 79 | 1 | 854 |
| 81 |  | 592 |
| 93 |  | 862 |
| 107 | 1 | 793 |
| 121 |  | 678 |
| 136. |  | 502 |

Collision Energy

0

Ionization Mode

EI

**Peak List**

| **m/z** | **Abund** |
| --- | --- |
| 40.9 | 112 |
| 67 | 213 |
| 95 | 174 |

Collision Energy

0

Ionization Mode

EI

**Peak List**

| **m/z** | **Abund** |
| --- | --- |
| 41.1 | 171 |
| 43 | 111 |
| 52.9 | 117 |
| 55 | 206 |
| 67 | 105 |
| 69 | 140 |
| 83 | 194 |
| 84 | 349 |
| 91.1 | 138 |
| 109 | 443 |

Collision Energy

0

Ionization Mode

EI

**Peak List**

| **m/z** | **z** | **Abund** |
| --- | --- | --- |
| 41 |  | 289 |
| 42 |  | 116 |
| 43 |  | 139 |
| 55 |  | 211 |
| 57 |  | 469 |
| 67 |  | 758 |
| 82 | 1 | 745 |
| 83 | 1 | 129 |
| 85 |  | 395 |

Collision Energy

0

Ionization Mode

EI

**Peak List**

| **m/z** | **Abund** |
| --- | --- |
| 41 | 187 |
| 43 | 202 |
| 55.1 | 169 |
| 56 | 168 |
| 57.1 | 142 |
| 68.9 | 140 |
| 83.1 | 118 |
| 84 | 346 |
| 85 | 197 |
| 108.9 | 127 |

Collision Energy

0

Ionization Mode

EI

**Peak List**

| **m/z** | **Abund** |
| --- | --- |
| 41.1 | 249 |
| 43 | 716 |
| 54.9 | 267 |
| 67 | 556 |
| 68.1 | 215 |
| 79 | 242 |
| 81.1 | 224 |
| 84.9 | 399 |
| 95 | 277 |
| 123 | 324 |

Collision Energy

0

Ionization Mode

EI

**Peak List**

| **m/z** | **Abund** |
| --- | --- |
| 41 | 223 |
| 43.1 | 331 |
| 54.9 | 189 |
| 69 | 561 |
| 78.9 | 141 |
| 84 | 201 |
| 90. | 226 |
| 95 | 182 |
| 107 | 344 |
| 179.1 | 653 |

Collision Energy

0

Ionization Mode

EI

**Peak List**

| **m/z** | **z** | **Abund** |
| --- | --- | --- |
| 40.9 |  | 278 |
| 43 |  | 335 |
| 55.1 |  | 336 |
| 69 |  | 561 |
| 79.1 |  | 184 |
| 91 |  | 269 |
| 95 |  | 197 |
| 106.9 |  | 278 |

| **m/z** | **z** | **Abund** |
| --- | --- | --- |
| 179.1 | 1 | 1484 |
| 180.1 | 1 | 196 |

Collision Energy

0

Ionization Mode

EI

**Peak List**

| **m/z** | **Abund** |
| --- | --- |
| 55 | 227 |
| 67 | 255 |
| 82.1 | 242 |
| 83 | 241 |

Collision Energy

0

Ionization Mode

EI

**Peak List**

| **m/z** | **Abund** |
| --- | --- |
| 41 | 109 |
| 42.9 | 160 |
| 84 | 140 |
| 90.9 | 112 |
| 109 | 103 |
| 119 | 120 |

Collision Energy

0

Ionization Mode

EI

**Peak List**

| **m/z** | **Abund** |
| --- | --- |
| 43 | 601 |
| 55 | 199 |
| 76.9 | 198 |
| 84.1 | 553 |
| 91 | 387 |
| 92.9 | 187 |
| 109 | 366 |
| 119.1 | 424 |
| 134 | 221 |
| 152. | 229 |

Collision Energy

0

Ionization Mode

EI

**Peak List**

| **m/z** | **z** | **Abund** |
| --- | --- | --- |
| 41 |  | 656 |
| 53 |  | 695 |
| 67 |  | 1195 |
| 68.1 |  | 932 |
| 79 |  | 1073 |
| 80 |  | 882 |
| 81 | 1 | 1983 |
| 91 |  | 694 |
| 93 |  | 1136 |
| 107 |  | 658 |

Collision Energy

0

Ionization Mode

EI

**Peak List**

| **m/z** | **Abund** |
| --- | --- |
| 55 | 363 |
| 77 | 326 |
| 79 | 590 |
| 91 | 351 |
| 93 | 292 |
| 110 | 362 |
| 122 | 317 |
| 135 | 243 |
| 149 | 268 |
| 164 | 338 |

Collision Energy

0

Ionization Mode

EI

**Peak List**

| **m/z** | **Abund** |
| --- | --- |
| 41 | 125 |
| 43 | 175 |
| 70.9 | 161 |
| 78.8 | 142 |
| 90.8 | 179 |
| 105 | 152 |
| 107 | 289 |
| 119.1 | 170 |
| 121 | 195 |
| 136 | 137 |

Collision Energy

0

Ionization Mode

EI

**Peak List**

| **m/z** | **Abund** |
| --- | --- |
| 41.1 | 203 |
| 69 | 223 |
| 76.9 | 146 |
| 79 | 213 |
| 81 | 116 |
| 90.9 | 229 |
| 93 | 233 |
| 105 | 152 |
| 107.1 | 114 |
| 133.1 | 167 |

Collision Energy

0

Ionization Mode

EI

**Peak List**

| **m/z** | **z** | **Abund** |
| --- | --- | --- |
| 41.1 |  | 244 |
| 55 |  | 195 |
| 77 |  | 261 |
| 79 |  | 276 |
| 81 |  | 226 |
| 91 |  | 450 |
| 93 |  | 247 |
| 105.1 |  | 407 |

| **m/z** | **z** | **Abund** |
| --- | --- | --- |
| 118.9 |  | 228 |
| 161.1 | 1 | 937 |

Collision Energy

0

Ionization Mode

EI

**Peak List**

| **m/z** | **Abund** |
| --- | --- |
| 41.1 | 328 |
| 53 | 114 |
| 55 | 125 |
| 66.9 | 199 |
| 69.1 | 651 |
| 78.9 | 130 |
| 81 | 138 |
| 91 | 123 |
| 92.9 | 326 |
| 133 | 136 |

Collision Energy

0

Ionization Mode

EI

**Peak List**

| **m/z** | **z** | **Abund** |
| --- | --- | --- |
| 41 |  | 303 |
| 55 |  | 243 |
| 77 |  | 326 |
| 79.1 |  | 370 |
| 81.1 |  | 401 |
| 91.1 |  | 507 |

| **m/z** | **z** | **Abund** |
| --- | --- | --- |
| 93 |  | 384 |
| 105 | 1 | 564 |
| 119 |  | 334 |
| 161.1 | 1 | 727 |

Collision Energy

0

Ionization Mode

EI

**Peak List**

| **m/z** | **z** | **Abund** |
| --- | --- | --- |
| 41.1 |  | 225 |
| 53 |  | 191 |
| 67 |  | 222 |
| 76.9 |  | 218 |
| 78.9 |  | 232 |
| 80.1 | 1 | 430 |
| 91 |  | 220 |
| 92 |  | 186 |
| 93 | 1 | 1096 |
| 121.1 |  | 278 |

Collision Energy

0

Ionization Mode

EI

**Peak List**

| **m/z** | **z** | **Abund** |
| --- | --- | --- |
| 41 |  | 280 |
| 76.9 |  | 237 |
| 79 |  | 256 |
| 81 |  | 281 |

| **m/z** | **z** | **Abund** |
| --- | --- | --- |
| 91 | 1 | 642 |
| 105 |  | 601 |
| 119 |  | 354 |
| 161.1 | 1 | 2011 |
| 162.1 | 1 | 269 |
| 204.1 |  | 322 |

Collision Energy

0

Ionization Mode

EI

**Peak List**

| **m/z** | **Abund** |
| --- | --- |
| 41.1 | 200 |
| 67 | 159 |
| 76.9 | 254 |
| 79 | 270 |
| 81.1 | 196 |
| 90.9 | 367 |
| 93 | 162 |
| 105.1 | 304 |
| 119 | 201 |
| 161 | 540 |

Collision Energy

0

Ionization Mode

EI

**Peak List**

| **m/z** | **Abund** |
| --- | --- |
| 41 | 201 |
| 77 | 141 |

| **m/z** | **Abund** |
| --- | --- |
| 79.1 | 251 |
| 81.1 | 157 |
| 91 | 271 |
| 92.9 | 177 |
| 105 | 257 |
| 119 | 222 |
| 161.1 | 583 |
| 204.1 | 123 |

Collision Energy

0

Ionization Mode

EI

**Peak List**

| **m/z** | **z** | **Abund** |
| --- | --- | --- |
| 105 |  | 145 |
| 115 |  | 196 |
| 128 |  | 276 |
| 129 |  | 295 |
| 131 |  | 189 |
| 143.1 |  | 130 |
| 143.9 |  | 164 |
| 159.1 | 1 | 2231 |
| 160.1 | 1 | 297 |
| 202 |  | 219 |

Collision Energy

0

Ionization Mode

EI

# Peak List

| **m/z** | **Abund** |
| --- | --- |
| 81.1 | 112 |
| 105.1 | 212 |

Collision Energy

0

Ionization Mode

EI

**Peak List**

| **m/z** | **Abund** |
| --- | --- |
| 41 | 103 |
| 55.1 | 100 |

Collision Energy

0

Ionization Mode

EI

**Peak List**

| **m/z** | **Abund** |
| --- | --- |
| 42.9 | 131 |
| 55 | 108 |

Collision Energy

0

Ionization Mode

EI

**Peak List**

| **m/z** | **Abund** |
| --- | --- |
| 41 | 116 |
| 43 | 208 |
| 54.9 | 110 |
| 69 | 231 |
| 81 | 115 |

Collision Energy

0

Ionization Mode

EI

**Peak List**

| **m/z** | **z** | **Abund** |
| --- | --- | --- |
| 41.1 |  | 332 |
| 43 |  | 351 |
| 55 |  | 390 |
| 78.9 |  | 288 |
| 81.1 |  | 355 |
| 95 |  | 326 |
| 105 |  | 387 |
| 119 | 1 | 606 |
| 161.1 |  | 585 |
| 179.1 |  | 475 |

Collision Energy

0

Ionization Mode

EI

**Peak List**

| **m/z** | **z** | **Abund** |
| --- | --- | --- |
| 41 |  | 256 |
| 43 |  | 515 |
| 71 |  | 237 |
| 78.9 |  | 315 |
| 81 |  | 286 |
| 91 |  | 221 |
| 93 |  | 213 |
| 95 | 1 | 766 |
| 104.9 |  | 222 |
| 121 | 1 | 488 |

Collision Energy

0

Ionization Mode

EI

**Peak List**

| **m/z** | **Abund** |
| --- | --- |
| 41 | 142 |
| 42.9 | 112 |
| 55 | 150 |
| 66.9 | 149 |
| 79 | 137 |
| 81.1 | 145 |
| 90.9 | 116 |
| 93.1 | 153 |
| 95 | 120 |

Collision Energy

0

Ionization Mode

EI

**Peak List**

| **m/z** | **Abund** |
| --- | --- |
| 41 | 178 |
| 42.9 | 149 |
| 55 | 191 |
| 66.9 | 133 |
| 77 | 111 |
| 78.9 | 165 |
| 81 | 119 |
| 90.9 | 170 |
| 93.1 | 168 |
| 107 | 123 |

Collision Energy

0

Ionization Mode

EI

**Peak List**

| **m/z** | **Abund** |
| --- | --- |
| 41 | 259 |
| 43 | 264 |
| 55.1 | 238 |
| 67 | 176 |
| 77 | 212 |
| 79 | 267 |
| 81 | 205 |
| 91 | 287 |
| 92.9 | 189 |
| 109 | 271 |

Collision Energy

0

Ionization Mode

EI

**Peak List**

| **m/z** | **Abund** |
| --- | --- |
| 43.1 | 104 |
| 55.1 | 102 |
| 57.2 | 127 |
| 96.9 | 181 |

Collision Energy

0

Ionization Mode

EI

**Peak List**

| **m/z** | **Abund** |
| --- | --- |
| 55 | 106 |

Collision Energy

0

Ionization Mode

EI

Spectrum Source

Peak (11) in "+ TIC Scan"

**Peak List**

| **m/z** | **z** | **Abund** |
| --- | --- | --- |
| 53.1 |  | 15333 |
| 67.1 |  | 50623 |
| 68.1 | 1 | 67485 |
| 77 |  | 13241 |
| 79.1 |  | 22752 |
| 91 |  | 13499 |
| 92 |  | 13578 |
| 93.1 |  | 42925 |
| 94.1 |  | 15582 |
| 121.1 |  | 12788 |

Spectrum Source

Peak (12) in "+ TIC Scan"

Collision Energy

0

Collision Energy

0

Ionization Mode

EI

Ionization Mode

EI

**Peak List**

| **m/z** | **z** | **Abund** |
| --- | --- | --- |
| 41.1 | 1 | 1296 |
| 43.1 |  | 3083 |
| 55.1 | 1 | 1373 |
| 69 |  | 1319 |
| 71 |  | 1512 |
| 79 |  | 1415 |
| 81.1 | 1 | 2009 |
| 91 |  | 1358 |
| 93 | 1 | 3672 |
| 108. |  | 1313 |

Spectrum Source

Peak (29) in "+ TIC Scan"

Collision Energy

0

Ionization Mode

EI

**Peak List**

| **m/z** | **z** | **Abund** |
| --- | --- | --- |
| 41.1 |  | 27651 |
| 53 |  | 36244 |
| 54.1 |  | 99321 |
| 79 |  | 35337 |
| 82 | 1 | 271124 |
| 91 |  | 31337 |
| 93.1 | 1 | 84726 |
| 106.1 |  | 36286 |
| 107.1 |  | 50298 |
| 108.1 |  | 83771 |

Spectrum Source

+ TIC Scan

Collision Energy

0

Ionization Mode

EI

**Peak List**

| **m/z** | **z** | **Abund** |
| --- | --- | --- |
| 41.1 |  | 1670 |
| 53 |  | 1017 |
| 77 |  | 2065 |
| 79 |  | 4574 |
| 80 |  | 9300 |
| 81.1 | 1 | 12281 |
| 91 |  | 1962 |
| 105 |  | 1431 |
| 123.1 | 1 | 7468 |
| 161.1 |  | 3024 |

Spectrum Source

Peak (42) in "+ TIC Scan"

Collision Energy

0

Ionization Mode

EI

**Peak List**

| **m/z** | **z** | **Abund** |
| --- | --- | --- |
| 41.1 | 1 | 5778 |
| 67 | 1 | 3591 |
| 69.1 | 1 | 6923 |
| 77 |  | 3804 |
| 79 |  | 6266 |
| 91 |  | 6323 |
| 93 | 1 | 7374 |
| 105 |  | 3800 |
| 107 | 1 | 3108 |
| 133.1 | 1 | 5420 |

Spectrum Source

Peak (49) in "+ TIC Scan"

Collision Energy

0

Ionization Mode

EI

**Peak List**

| **m/z** | **z** | **Abund** |
| --- | --- | --- |
| 41.1 |  | 1115 |
| 77 |  | 1318 |
| 79 |  | 1463 |
| 81.1 | 1 | 1494 |
| 91 | 1 | 2166 |
| 93.1 | 1 | 1008 |
| 105. | 1 | 2085 |
| 119 |  | 1209 |
| 120.1 | 1 | 882 |
| 161.1 | 1 | 3321 |

Collision Energy

0

Ionization Mode

EI

**Peak List**

| **m/z** | **Abund** |
| --- | --- |
| 39 | 117 |
| 41.1 | 304 |
| 42.1 | 204 |
| 55 | 310 |
| 57 | 202 |
| 69 | 275 |
| 70 | 100 |
| 83.1 | 205 |

Collision Energy

0

Ionization Mode

EI

**Peak List**

| **m/z** | **Abund** |
| --- | --- |
| 41 | 132 |
| 42 | 127 |
| 43 | 156 |
| 55 | 398 |
| 57 | 172 |
| 70 | 129 |
| 81 | 554 |
| 99 | 327 |

Collision Energy

0

Ionization Mode

EI

**Peak List**

| **m/z** | **z** | **Abund** |
| --- | --- | --- |
| 41.1 |  | 453 |
| 77 |  | 1178 |
| 79 |  | 1017 |
| 80 |  | 367 |
| 91 |  | 1533 |
| 92 |  | 1235 |
| 93.1 | 1 | 3681 |
| 94.1 | 1 | 374 |
| 105 |  | 365 |
| 121 |  | 378 |

Collision Energy

0

Ionization Mode

EI

**Peak List**

| **m/z** | **z** | **Abund** |
| --- | --- | --- |
| 41.1 |  | 440 |
| 69 |  | 263 |
| 77 | 1 | 1044 |
| 79 |  | 716 |
| 80 |  | 275 |
| 91 |  | 1121 |
| 92 |  | 295 |
| 93 | 1 | 2400 |
| 94 | 1 | 289 |
| 136 |  | 319 |

Collision Energy

0

Ionization Mode

EI

**Peak List**

| **m/z** | **z** | **Abund** |
| --- | --- | --- |
| 41.1 |  | 1945 |
| 53 |  | 726 |
| 67.1 |  | 788 |
| 69.1 |  | 2328 |
| 77 |  | 1654 |
| 79 |  | 1642 |
| 80.1 |  | 766 |
| 91 |  | 1790 |
| 93.1 | 1 | 6232 |
| 94.1 | 1 | 819 |

Collision Energy

0

Ionization Mode

EI

**Peak List**

| **m/z** | **z** | **Abund** |
| --- | --- | --- |
| 41.1 |  | 3364 |
| 53.1 |  | 733 |
| 67 |  | 640 |
| 69 |  | 4030 |
| 76.9 |  | 759 |
| 79.1 |  | 817 |
| 91 |  | 1053 |
| 92 |  | 459 |
| 93.1 | 1 | 4453 |
| 94.1 | 1 | 475 |

Collision Energy

0

Ionization Mode

EI

**Peak List**

| **m/z** | **z** | **Abund** |
| --- | --- | --- |
| 41.1 |  | 1602 |
| 43 |  | 846 |
| 44 |  | 506 |
| 55 | 1 | 3668 |
| 56.1 | 1 | 471 |
| 57.1 |  | 946 |
| 58 |  | 599 |
| 59.1 |  | 7067 |

| **m/z** | **z** | **Abund** |
| --- | --- | --- |
| 83.1 |  | 3611 |
| 101.1 |  | 1511 |

Collision Energy

0

Ionization Mode

EI

**Peak List**

| **m/z** | **Abund** |
| --- | --- |
| 41.1 | 166 |
| 43 | 180 |
| 44.1 | 140 |
| 55.1 | 171 |
| 56 | 163 |
| 56.9 | 205 |

Collision Energy

0

Ionization Mode

EI

**Peak List**

| **m/z** | **Abund** |
| --- | --- |
| 41 | 117 |
| 79 | 140 |
| 91.1 | 128 |
| 93 | 294 |

Collision Energy

0

Ionization Mode

EI

**Peak List**

| **m/z** | **z** | **Abund** |
| --- | --- | --- |
| 41.1 |  | 542 |
| 52.9 |  | 416 |
| 67 |  | 362 |
| 77 | 1 | 782 |
| 79 |  | 1039 |
| 80.1 | 1 | 805 |
| 91 |  | 1173 |
| 92 |  | 514 |
| 93 | 1 | 2128 |
| 105 |  | 361 |

Collision Energy

0

Ionization Mode

EI

**Peak List**

| **m/z** | **Abund** |
| --- | --- |
| 40.9 | 202 |
| 43.1 | 164 |
| 55 | 575 |
| 57 | 159 |
| 59 | 821 |
| 69 | 121 |
| 97 | 395 |
| 115 | 188 |

Collision Energy

0

Ionization Mode

EI

**Peak List**

| **m/z** | **Abund** |
| --- | --- |
| 41 | 416 |
| 42 | 146 |
| 43.1 | 558 |
| 55 | 466 |
| 56 | 133 |
| 57 | 953 |
| 70 | 1302 |
| 71 | 348 |
| 85 | 605 |
| 103 | 248 |

Collision Energy

0

Ionization Mode

EI

**Peak List**

| **m/z** | **Abund** |
| --- | --- |
| 41 | 368 |
| 43.1 | 422 |
| 44 | 157 |
| 55 | 362 |
| 56 | 222 |
| 57.1 | 878 |
| 69 | 144 |
| 70 | 928 |
| 71 | 319 |
| 85 | 713 |

Collision Energy

0

Ionization Mode

EI

**Peak List**

| **m/z** | **Abund** |
| --- | --- |
| 41 | 568 |
| 43 | 4424 |
| 55.1 | 609 |
| 56 | 373 |
| 57 | 361 |
| 69.1 | 269 |
| 70 | 443 |
| 83 | 543 |
| 101 | 886 |
| 112 | 353 |

Collision Energy

0

Ionization Mode

EI

**Peak List**

| **m/z** | **Abund** |
| --- | --- |
| 43.1 | 145 |
| 79 | 153 |
| 94 | 117 |
| 108.9 | 125 |

Collision Energy

0

Ionization Mode

EI

**Peak List**

| **m/z** | **Abund** |
| --- | --- |
| 41.1 | 317 |
| 43.1 | 374 |
| 57 | 354 |
| 67 | 339 |
| 68.1 | 217 |
| 69.1 | 243 |
| 78.9 | 184 |
| 93 | 143 |
| 94 | 236 |
| 108. | 146 |

Collision Energy

0

Ionization Mode

EI

**Peak List**

| **m/z** | **Abund** |
| --- | --- |
| 41 | 253 |
| 43 | 402 |
| 54.9 | 254 |
| 66.9 | 253 |
| 68 | 170 |
| 69 | 262 |
| 71 | 906 |
| 86 | 234 |
| 93 | 380 |
| 111 | 503 |

Collision Energy

0

Ionization Mode

EI

**Peak List**

| **m/z** | **Abund** |
| --- | --- |
| 40.9 | 129 |
| 54.9 | 132 |
| 59 | 165 |
| 69 | 192 |

Collision Energy

0

Ionization Mode

EI

**Peak List**

| **m/z** | **Abund** |
| --- | --- |
| 40.9 | 132 |
| 43 | 296 |
| 55 | 121 |
| 59 | 728 |
| 66.9 | 179 |
| 79 | 174 |
| 81 | 271 |
| 93.1 | 372 |
| 121.1 | 295 |
| 136.1 | 227 |

Collision Energy

0

Ionization Mode

EI

**Peak List**

| **m/z** | **z** | **Abund** |
| --- | --- | --- |
| 41 | 1 | 874 |
| 43.1 | 1 | 724 |
| 55.1 |  | 881 |
| 67.1 |  | 1020 |
| 79 | 1 | 1159 |
| 81 |  | 711 |
| 93 |  | 1336 |
| 107.1 |  | 1218 |
| 121 |  | 1089 |
| 136 | 1 | 767 |

Collision Energy

0

Ionization Mode

EI

**Peak List**

| **m/z** | **z** | **Abund** |
| --- | --- | --- |
| 41.1 |  | 2359 |
| 53 |  | 1316 |
| 55 |  | 2098 |
| 67 |  | 5983 |
| 68 |  | 3008 |
| 69.1 | 1 | 2190 |
| 81.1 |  | 2539 |
| 82.1 | 1 | 2592 |
| 95.1 | 1 | 3627 |
| 109 |  | 1629 |

Collision Energy

0

Ionization Mode

EI

**Peak List**

| **m/z** | **Abund** |
| --- | --- |
| 41 | 209 |
| 54.9 | 145 |
| 67 | 431 |
| 68.1 | 203 |
| 69 | 180 |
| 79 | 109 |
| 81.1 | 136 |
| 82 | 175 |
| 95 | 293 |
| 152 | 104 |

Collision Energy

0

Ionization Mode

EI

**Peak List**

| **m/z** | **Abund** |
| --- | --- |
| 41.1 | 280 |
| 53 | 206 |
| 55 | 345 |
| 69 | 265 |
| 76.9 | 204 |
| 83 | 285 |
| 84.1 | 554 |
| 91 | 220 |
| 95 | 208 |
| 109.1 | 804 |

Collision Energy

0

Ionization Mode

EI

**Peak List**

| **m/z** | **z** | **Abund** |
| --- | --- | --- |
| 41.1 |  | 458 |
| 42 |  | 159 |
| 43 |  | 259 |
| 55 |  | 363 |
| 57.1 |  | 824 |
| 67 | 1 | 1291 |
| 69. | 1 | 118 |
| 82.1 | 1 | 1313 |
| 83.1 | 1 | 238 |
| 85 |  | 746 |

Collision Energy

0

Ionization Mode

EI

**Peak List**

| **m/z** | **Abund** |
| --- | --- |
| 41 | 350 |
| 43 | 361 |
| 55.1 | 360 |
| 56.1 | 316 |
| 57.1 | 253 |
| 69 | 354 |
| 83 | 265 |
| 84 | 659 |

| **m/z** | **Abund** |
| --- | --- |
| 85 | 371 |
| 109 | 310 |

Collision Energy

0

Ionization Mode

EI

**Peak List**

| **m/z** | **Abund** |
| --- | --- |
| 41 | 507 |
| 43 | 1634 |
| 52.9 | 531 |
| 55 | 646 |
| 67 | 1145 |
| 68 | 472 |
| 79 | 461 |
| 85 | 958 |
| 95 | 655 |
| 123 | 669 |

Collision Energy

0

Ionization Mode

EI

**Peak List**

| **m/z** | **z** | **Abund** |
| --- | --- | --- |
| 41.1 |  | 295 |
| 43.1 |  | 398 |
| 55.1 |  | 280 |
| 69 |  | 679 |
| 79 |  | 183 |
| 84 |  | 343 |

| **m/z** | **z** | **Abund** |
| --- | --- | --- |
| 90.9 |  | 288 |
| 95 |  | 227 |
| 107 |  | 391 |
| 179.1 | 1 | 821 |

Collision Energy

0

Ionization Mode

EI

**Peak List**

| **m/z** | **z** | **Abund** |
| --- | --- | --- |
| 41.1 |  | 592 |
| 43.1 |  | 664 |
| 55 |  | 599 |
| 69 |  | 1136 |
| 77 |  | 350 |
| 91 |  | 554 |
| 95 |  | 420 |
| 107 |  | 560 |
| 179.1 | 1 | 2943 |
| 180.1 | 1 | 393 |

Collision Energy

0

Ionization Mode

EI

**Peak List**

| **m/z** | **z** | **Abund** |
| --- | --- | --- |
| 41.1 | 1 | 869 |
| 43.1 | 1 | 3019 |
| 67.1 |  | 888 |
| 68.1 |  | 845 |

| **m/z** | **z** | **Abund** |
| --- | --- | --- |
| 79 |  | 1256 |
| 93 |  | 2051 |
| 94.1 |  | 855 |
| 107.1 | 1 | 1700 |
| 121.1 | 1 | 1625 |
| 136.1 |  | 1188 |

Collision Energy

0

Ionization Mode

EI

**Peak List**

| **m/z** | **Abund** |
| --- | --- |
| 43.1 | 694 |
| 54.9 | 258 |
| 76.9 | 300 |
| 78.9 | 251 |
| 84 | 697 |
| 91.1 | 453 |
| 109 | 451 |
| 119 | 533 |
| 134 | 277 |
| 152 | 297 |

Collision Energy

0

Ionization Mode

EI

**Peak List**

| **m/z** | **z** | **Abund** |
| --- | --- | --- |
| 41.1 | 1 | 1359 |
| 53 |  | 1439 |

| **m/z** | **z** | **Abund** |
| --- | --- | --- |
| 67 |  | 2158 |
| 68.1 |  | 1998 |
| 79 |  | 2013 |
| 80 |  | 1353 |
| 81 | 1 | 3623 |
| 91 |  | 1379 |
| 93.1 |  | 2342 |
| 107.1 |  | 1522 |

Collision Energy

0

**Peak List**

Collision Energy

| **m/z** | **z** | **Abund** |
| --- | --- | --- |
| 55 |  | 554 |
| 66.9 |  | 406 |
| 77 |  | 512 |
| 79 | 1 | 905 |
| 91 | 1 | 625 |
| 93 | 1 | 422 |
| 110 |  | 596 |
| 122 | 1 | 409 |
| 149 |  | 435 |
| 164.1 |  | 583 |

0

Ionization Mode

EI

Ionization Mode

EI

# Peak List

| **m/z** | **Abund** |
| --- | --- |
| 43.1 | 266 |
| 71 | 202 |
| 76.9 | 159 |
| 79 | 211 |
| 91 | 306 |
| 93 | 216 |
| 105 | 204 |
| 107 | 374 |
| 119 | 227 |
| 121.1 | 278 |

Collision Energy

0

Ionization Mode

EI

**Peak List**

| **m/z** | **Abund** |
| --- | --- |
| 41 | 385 |
| 55 | 200 |
| 67 | 231 |
| 69 | 333 |
| 77 | 229 |
| 78.9 | 420 |
| 91 | 352 |
| 93 | 408 |
| 104.9 | 237 |
| 133 | 266 |

Collision Energy

0

Ionization Mode

EI

**Peak List**

| **m/z** | **z** | **Abund** |
| --- | --- | --- |
| 41.1 |  | 369 |
| 77 |  | 344 |
| 79.1 |  | 351 |
| 81 |  | 317 |
| 91 | 1 | 642 |
| 93 | 1 | 276 |
| 105 | 1 | 640 |
| 118.9 |  | 314 |
| 120 |  | 273 |
| 161. | 1 | 1160 |

Collision Energy

0

Ionization Mode

EI

**Peak List**

| **m/z** | **z** | **Abund** |
| --- | --- | --- |
| 41.1 |  | 736 |
| 53 |  | 275 |
| 55 |  | 271 |
| 67 |  | 412 |
| 69.1 | 1 | 1359 |
| 79 |  | 316 |
| 81 |  | 279 |
| 91 |  | 227 |
| 93 | 1 | 697 |
| 133 |  | 281 |

Collision Energy

0

Ionization Mode

EI

**Peak List**

| **m/z** | **z** | **Abund** |
| --- | --- | --- |
| 41.1 |  | 474 |
| 55.1 |  | 365 |
| 77 | 1 | 564 |
| 79.1 |  | 595 |
| 81 |  | 471 |
| 91 | 1 | 783 |
| 93 | 1 | 467 |
| 105 | 1 | 816 |
| 119 |  | 398 |
| 161.1 | 1 | 883 |

Collision Energy

0

Ionization Mode

EI

**Peak List**

| **m/z** | **z** | **Abund** |
| --- | --- | --- |
| 41.1 |  | 309 |
| 55 |  | 291 |
| 67 |  | 376 |
| 77 |  | 346 |
| 79 |  | 380 |
| 80 |  | 670 |
| 91 |  | 386 |
| 92 |  | 311 |
| 93 | 1 | 1816 |
| 121.1 | 1 | 493 |

Collision Energy

0

Ionization Mode

EI

**Peak List**

| **m/z** | **z** | **Abund** |
| --- | --- | --- |
| 41.1 |  | 532 |
| 79 |  | 444 |
| 81 |  | 477 |
| 91 |  | 1149 |
| 93 |  | 451 |
| 105.1 |  | 1075 |
| 119 |  | 645 |
| 161.1 | 1 | 3229 |
| 162.1 | 1 | 475 |
| 204.1 |  | 452 |

Collision Energy

0

Ionization Mode

EI

**Peak List**

| **m/z** | **z** | **Abund** |
| --- | --- | --- |
| 41 |  | 252 |
| 55 |  | 180 |
| 66.9 |  | 179 |
| 76.9 |  | 257 |
| 79 |  | 316 |
| 81 |  | 292 |
| 90.9 |  | 409 |
| 105 |  | 368 |

| **m/z** | **z** | **Abund** |
| --- | --- | --- |
| 119 |  | 214 |
| 161.1 | 1 | 603 |

Collision Energy

0

Ionization Mode

EI

**Peak List**

| **m/z** | **z** | **Abund** |
| --- | --- | --- |
| 41.1 |  | 293 |
| 55 |  | 203 |
| 77 |  | 227 |
| 79 |  | 284 |
| 81 |  | 207 |
| 91.1 | 1 | 406 |
| 93 | 1 | 281 |
| 105 |  | 402 |
| 119.1 |  | 336 |
| 161.1 | 1 | 752 |

Collision Energy

0

Ionization Mode

EI

**Peak List**

| **m/z** | **z** | **Abund** |
| --- | --- | --- |
| 105 |  | 150 |
| 115 |  | 229 |
| 127.9 |  | 290 |
| 129 |  | 338 |
| 131 |  | 257 |
| 143.1 |  | 173 |

| **m/z** | **z** | **Abund** |
| --- | --- | --- |
| 144 |  | 230 |
| 159.1 | 1 | 2863 |
| 160.1 | 1 | 364 |
| 202.1 |  | 321 |

Collision Energy

0

Ionization Mode

EI

**Peak List**

| **m/z** | **Abund** |
| --- | --- |
| 41 | 126 |
| 77 | 100 |
| 81 | 174 |
| 90.9 | 163 |
| 93.1 | 123 |
| 105 | 459 |
| 161 | 189 |

Collision Energy

0

Ionization Mode

EI

**Peak List**

| **m/z** | **Abund** |
| --- | --- |
| 41 | 130 |
| 43.1 | 103 |
| 55 | 150 |
| 137.1 | 120 |
| 207.1 | 125 |

Collision Energy

0

Ionization Mode

EI

**Peak List**

| **m/z** | **Abund** |
| --- | --- |
| 43.1 | 243 |
| 55 | 112 |
| 79 | 111 |
| 84.1 | 218 |
| 91.1 | 150 |
| 92 | 103 |
| 104.9 | 106 |
| 109 | 198 |
| 119 | 232 |
| 152 | 154 |

Collision Energy

0

Ionization Mode

EI

**Peak List**

| **m/z** | **Abund** |
| --- | --- |
| 40.9 | 101 |
| 55 | 400 |
| 67 | 411 |
| 82.1 | 414 |
| 83 | 440 |

Collision Energy

0

Ionization Mode

EI

**Peak List**

| **m/z** | **Abund** |
| --- | --- |
| 41.1 | 141 |
| 42.9 | 271 |
| 55.1 | 161 |
| 57.1 | 129 |
| 58.1 | 146 |
| 69.1 | 116 |
| 71.1 | 120 |

Collision Energy

0

Ionization Mode

EI

**Peak List**

| **m/z** | **Abund** |
| --- | --- |
| 41 | 149 |
| 43 | 197 |
| 55.1 | 127 |
| 69 | 210 |
| 80.9 | 106 |

Collision Energy

0

Ionization Mode

EI

**Peak List**

| **m/z** | **z** | **Abund** |
| --- | --- | --- |
| 41 | 1 | 2215 |
| 43.1 | 1 | 2515 |
| 55.1 |  | 1375 |
| 67 |  | 1468 |
| 69 | 1 | 1903 |
| 77 |  | 1348 |
| 79 | 1 | 2650 |
| 91 | 1 | 1933 |
| 93.1 | 1 | 1942 |
| 95 |  | 1284 |

Collision Energy

0

Ionization Mode

EI

**Peak List**

| **m/z** | **z** | **Abund** |
| --- | --- | --- |
| 41 |  | 480 |
| 43 |  | 432 |
| 55 |  | 464 |
| 81 |  | 435 |
| 95 |  | 403 |
| 105 |  | 419 |
| 119 | 1 | 770 |
| 161.1 | 1 | 688 |
| 179.1 |  | 500 |
| 204.2 |  | 355 |

Collision Energy

0

Ionization Mode

EI

**Peak List**

| **m/z** | **z** | **Abund** |
| --- | --- | --- |
| 41.1 |  | 1067 |
| 43.1 |  | 1473 |
| 55 |  | 836 |
| 79 | 1 | 1167 |
| 81 | 1 | 1147 |
| 91 |  | 891 |
| 95 | 1 | 1041 |
| 105 | 1 | 1132 |
| 161.1 | 1 | 3534 |
| 204.2 | 1 | 1263 |

Collision Energy

0

Ionization Mode

EI

**Peak List**

| **m/z** | **z** | **Abund** |
| --- | --- | --- |
| 41.1 |  | 277 |
| 43.1 |  | 622 |
| 54.9 |  | 266 |
| 79 |  | 345 |
| 81 |  | 360 |
| 95 | 1 | 858 |
| 105.1 |  | 264 |
| 121.1 |  | 603 |
| 161.1 |  | 273 |
| 204.1 |  | 266 |

Collision Energy

0

Ionization Mode

EI

**Peak List**

| **m/z** | **Abund** |
| --- | --- |
| 41 | 185 |
| 43 | 135 |
| 55.1 | 215 |
| 67 | 188 |
| 69.1 | 118 |
| 78.9 | 141 |
| 81 | 162 |
| 93 | 128 |
| 95.1 | 120 |

Collision Energy

0

Ionization Mode

EI

**Peak List**

| **m/z** | **Abund** |
| --- | --- |
| 41 | 189 |
| 43 | 156 |
| 54.9 | 159 |
| 67 | 140 |
| 69 | 128 |
| 76.8 | 133 |
| 78.9 | 164 |
| 81 | 122 |
| 91 | 165 |

| **m/z** | **Abund** |
| --- | --- |
| 93.1 | 130 |

Collision Energy

0

Ionization Mode

EI

**Peak List**

| **m/z** | **Abund** |
| --- | --- |
| 41 | 205 |
| 43.1 | 210 |
| 55.1 | 209 |
| 67 | 180 |
| 77 | 172 |
| 79 | 209 |
| 81 | 190 |
| 91 | 276 |
| 93 | 191 |
| 109 | 270 |

Collision Energy

0

Ionization Mode

EI

**Peak List**

| **m/z** | **Abund** |
| --- | --- |
| 41.1 | 109 |
| 42.9 | 131 |
| 55.1 | 113 |
| 57 | 132 |
| 97 | 144 |

Collision Energy

0

Ionization Mode

EI

**Peak List**

| **m/z** | **Abund** |
| --- | --- |
| 41.2 | 109 |
| 55.1 | 124 |
| 93.1 | 103 |

Collision Energy

0

Ionization Mode

EI

**Peak List**

| **m/z** | **Abund** |
| --- | --- |
| 41 | 138 |
| 67 | 109 |
| 68.9 | 103 |
| 93 | 209 |

Spectrum Source

Peak (11) in "+ TIC Scan"

Collision Energy

0

Ionization Mode

EI

**Peak List**

| **m/z** | **z** | **Abund** |
| --- | --- | --- |
| 53 |  | 11838 |
| 67.1 |  | 39039 |
| 68.1 | 1 | 51734 |
| 77 |  | 10036 |
| 79 |  | 17339 |
| 91 |  | 10301 |
| 92 |  | 10219 |
| 93.1 |  | 32090 |
| 94.1 |  | 11820 |
| 121. |  | 9567 |

Spectrum Source

Peak (29) in "+ TIC Scan"

Collision Energy

0

Ionization Mode

EI

**Peak List**

| **m/z** | **z** | **Abund** |
| --- | --- | --- |
| 41.1 |  | 24607 |
| 53 |  | 31889 |
| 54.1 |  | 86348 |
| 79.1 |  | 30493 |
| 82 | 1 | 231078 |
| 91 |  | 26867 |
| 93.1 | 1 | 71444 |
| 106.1 |  | 30426 |
| 107.1 |  | 42378 |
| 108.1 |  | 70533 |

Spectrum Source

Peak (30) in "+ TIC Scan"

Collision Energy

0

Ionization Mode

EI

**Peak List**

| **m/z** | **z** | **Abund** |
| --- | --- | --- |
| 41.1 |  | 794 |
| 53 |  | 539 |
| 54 |  | 754 |
| 55.1 |  | 553 |
| 82 | 1 | 4444 |
| 95 |  | 1510 |
| 109 |  | 831 |
| 110 | 1 | 3181 |
| 137.1 |  | 877 |
| 152.1 |  | 611 |

Spectrum Source

+ TIC Scan

Collision Energy

0

Ionization Mode

EI

**Peak List**

| **m/z** | **z** | **Abund** |
| --- | --- | --- |
| 41.1 |  | 1734 |
| 53 |  | 1111 |
| 77 |  | 2165 |
| 79 |  | 4721 |
| 80.1 |  | 9418 |
| 81.1 | 1 | 12597 |
| 91 |  | 1993 |
| 105 |  | 1440 |
| 123.1 | 1 | 7520 |
| 161.1 |  | 3014 |

Spectrum Source

Peak (43) in "+ TIC Scan"

Collision Energy

0

Ionization Mode

EI

**Peak List**

| **m/z** | **z** | **Abund** |
| --- | --- | --- |
| 41.1 | 1 | 5568 |
| 67.1 | 1 | 3483 |
| 69.1 | 1 | 6597 |
| 77 |  | 3645 |
| 79 |  | 5981 |
| 81.1 |  | 2977 |
| 91 |  | 6122 |
| 93 | 1 | 7124 |
| 105 |  | 3691 |
| 133.1 | 1 | 5232 |

Spectrum Source

Peak (50) in "+ TIC Scan"

Collision Energy

0

Ionization Mode

EI

**Peak List**

| **m/z** | **z** | **Abund** |
| --- | --- | --- |
| 41.1 |  | 1048 |
| 77 |  | 1168 |
| 79 |  | 1351 |
| 81.1 |  | 1377 |
| 91 | 1 | 2032 |
| 93.1 | 1 | 905 |
| 105.1 | 1 | 1907 |
| 119 |  | 1093 |

| **m/z** | **z** | **Abund** |
| --- | --- | --- |
| 120.1 | 1 | 808 |
| 161.1 | 1 | 2956 |

Spectrum Source

+ TIC Scan

Collision Energy

0

Ionization Mode

EI

**Peak List**

| **m/z** | **z** | **Abund** |
| --- | --- | --- |
| 41.1 |  | 1115 |
| 43.1 |  | 1305 |
| 55.1 |  | 721 |
| 67 | 1 | 695 |
| 69.1 |  | 949 |
| 77 |  | 689 |
| 79 | 1 | 1435 |
| 91 | 1 | 975 |
| 93 | 1 | 1037 |
| 95.1 |  | 693 |

Collision Energy

0

Ionization Mode

EI

**Peak List**

| **m/z** | **Abund** |
| --- | --- |
| 41.1 | 262 |
| 42 | 141 |
| 55 | 228 |
| 57 | 158 |
| 69 | 227 |
| 83 | 181 |

Collision Energy

0

Ionization Mode

EI

**Peak List**

| **m/z** | **Abund** |
| --- | --- |
| 42.1 | 124 |
| 43.1 | 147 |
| 55 | 378 |
| 57 | 179 |
| 70.1 | 134 |
| 81 | 579 |
| 99 | 336 |

Collision Energy

0

Ionization Mode

EI

**Peak List**

| **m/z** | **z** | **Abund** |
| --- | --- | --- |
| 41 |  | 405 |
| 53 |  | 299 |
| 67 |  | 309 |
| 77 | 1 | 901 |
| 79 |  | 685 |
| 80 |  | 299 |
| 91.1 |  | 1111 |
| 92 |  | 1055 |
| 93.1 | 1 | 2865 |
| 105 |  | 356 |

Collision Energy

0

Ionization Mode

EI

**Peak List**

| **m/z** | **z** | **Abund** |
| --- | --- | --- |
| 41 |  | 397 |
| 69 |  | 214 |
| 77 | 1 | 746 |
| 79 |  | 562 |
| 80 |  | 220 |
| 91 | 1 | 861 |
| 92 | 1 | 187 |
| 93.1 | 1 | 1879 |
| 94.1 | 1 | 236 |
| 136 |  | 246 |

Collision Energy

0

Ionization Mode

EI

**Peak List**

| **m/z** | **z** | **Abund** |
| --- | --- | --- |
| 41 |  | 1382 |
| 67 |  | 559 |
| 69.1 |  | 1667 |
| 77 |  | 1222 |
| 79 |  | 1122 |
| 80.1 |  | 492 |
| 91.1 |  | 1320 |
| 93 | 1 | 4441 |
| 94 | 1 | 591 |
| 121 |  | 521 |

Collision Energy

0

Ionization Mode

EI

**Peak List**

| **m/z** | **z** | **Abund** |
| --- | --- | --- |
| 39.1 |  | 244 |
| 41.1 |  | 1672 |
| 52.9 |  | 382 |
| 67 |  | 376 |
| 69.1 | 1 | 2019 |
| 76.9 |  | 403 |
| 79 |  | 391 |
| 91 |  | 597 |
| 92 |  | 267 |
| 93 | 1 | 2314 |

Collision Energy

0

Ionization Mode

EI

**Peak List**

| **m/z** | **z** | **Abund** |
| --- | --- | --- |
| 41.1 |  | 1250 |
| 43 |  | 761 |
| 44 |  | 427 |
| 55.1 | 1 | 2847 |
| 56 | 1 | 308 |
| 57 |  | 726 |
| 58 |  | 500 |
| 59 |  | 5575 |

| **m/z** | **z** | **Abund** |
| --- | --- | --- |
| 83.1 |  | 2924 |
| 101.1 |  | 1269 |

Collision Energy

0

Ionization Mode

EI

**Peak List**

| **m/z** | **Abund** |
| --- | --- |
| 41 | 217 |
| 43.2 | 208 |
| 44.1 | 176 |
| 54.9 | 164 |
| 56 | 163 |
| 57.1 | 190 |

Collision Energy

0

Ionization Mode

EI

**Peak List**

| **m/z** | **Abund** |
| --- | --- |
| 79 | 111 |
| 91 | 115 |
| 93 | 211 |

Collision Energy

0

Ionization Mode

EI

**Peak List**

| **m/z** | **z** | **Abund** |
| --- | --- | --- |
| 41 | 1 | 1276 |
| 43.1 |  | 2952 |
| 55.1 | 1 | 1311 |
| 67.1 |  | 1313 |
| 68 |  | 1246 |
| 71 |  | 1482 |
| 81.1 | 1 | 1891 |
| 84.1 |  | 1250 |
| 93 | 1 | 2972 |
| 108 | 1 | 1265 |

Collision Energy

0

Ionization Mode

EI

**Peak List**

| **m/z** | **z** | **Abund** |
| --- | --- | --- |
| 41 |  | 408 |
| 52.9 |  | 304 |
| 77 | 1 | 602 |
| 79 |  | 743 |
| 80.1 |  | 597 |
| 91 |  | 849 |
| 92 |  | 321 |
| 93 | 1 | 1565 |
| 105 |  | 253 |
| 121 |  | 262 |

Collision Energy

0

Ionization Mode

EI

**Peak List**

| **m/z** | **Abund** |
| --- | --- |
| 41 | 197 |
| 43 | 182 |
| 55.1 | 476 |
| 57 | 109 |
| 59 | 651 |
| 69 | 101 |
| 97 | 324 |
| 115 | 124 |

Collision Energy

0

Ionization Mode

EI

**Peak List**

| **m/z** | **Abund** |
| --- | --- |
| 41 | 377 |
| 42.1 | 117 |
| 43.1 | 522 |
| 55 | 458 |
| 56.1 | 121 |
| 57 | 873 |
| 70.1 | 1158 |
| 71 | 330 |
| 85 | 527 |
| 103.1 | 196 |

Collision Energy

0

Ionization Mode

EI

**Peak List**

| **m/z** | **Abund** |
| --- | --- |
| 41.1 | 351 |
| 43 | 424 |
| 44 | 204 |
| 55 | 334 |
| 56.1 | 230 |
| 57 | 676 |
| 69 | 155 |
| 70 | 606 |
| 71.1 | 194 |
| 85 | 344 |

Collision Energy

0

Ionization Mode

EI

**Peak List**

| **m/z** | **Abund** |
| --- | --- |
| 41 | 356 |
| 43.1 | 3158 |
| 55 | 371 |
| 56 | 309 |
| 57 | 284 |
| 69 | 192 |
| 70 | 307 |
| 83.1 | 320 |
| 101.1 | 616 |
| 112.1 | 248 |

Collision Energy

0

Ionization Mode

EI

**Peak List**

| **m/z** | **Abund** |
| --- | --- |
| 43 | 148 |
| 79.1 | 150 |
| 94 | 148 |
| 109 | 142 |

Collision Energy

0

Ionization Mode

EI

**Peak List**

| **m/z** | **Abund** |
| --- | --- |
| 41 | 262 |
| 42.9 | 338 |
| 55.1 | 152 |
| 57 | 269 |
| 67 | 367 |
| 69 | 203 |
| 79 | 188 |
| 80.9 | 140 |
| 94 | 226 |
| 108 | 166 |

Collision Energy

0

Ionization Mode

EI

**Peak List**

| **m/z** | **Abund** |
| --- | --- |
| 41.2 | 104 |
| 43 | 182 |
| 55.2 | 130 |
| 67 | 119 |
| 69 | 136 |
| 71 | 336 |
| 92.9 | 198 |
| 111 | 157 |

Collision Energy

0

Ionization Mode

EI

**Peak List**

| **m/z** | **Abund** |
| --- | --- |
| 41 | 105 |
| 55.1 | 101 |
| 59 | 158 |
| 69 | 149 |

Collision Energy

0

Ionization Mode

EI

**Peak List**

| **m/z** | **Abund** |
| --- | --- |
| 41 | 118 |
| 43 | 228 |
| 55 | 114 |
| 59 | 506 |
| 66.9 | 125 |
| 79 | 126 |
| 81 | 226 |
| 93.1 | 268 |
| 121 | 226 |
| 136. | 157 |

Collision Energy

0

Ionization Mode

EI

**Peak List**

| **m/z** | **z** | **Abund** |
| --- | --- | --- |
| 41 | 1 | 553 |
| 43.1 |  | 440 |
| 55 |  | 508 |
| 67 |  | 612 |
| 79 | 1 | 747 |
| 81 |  | 462 |
| 93 |  | 722 |
| 107 | 1 | 696 |
| 121.1 |  | 635 |
| 136.1 |  | 466 |

Collision Energy

0

Ionization Mode

EI

**Peak List**

| **m/z** | **z** | **Abund** |
| --- | --- | --- |
| 41.1 |  | 1374 |
| 53 |  | 751 |
| 55.1 |  | 1130 |
| 67 |  | 3206 |
| 68.1 |  | 1713 |
| 69 | 1 | 1236 |
| 81 |  | 1363 |
| 82 | 1 | 1425 |
| 95 | 1 | 2133 |
| 109.1 |  | 884 |

Collision Energy

0

Ionization Mode

EI

**Peak List**

| **m/z** | **Abund** |
| --- | --- |
| 40.9 | 103 |
| 67 | 176 |
| 68 | 101 |

Collision Energy

0

Ionization Mode

EI

**Peak List**

| **m/z** | **Abund** |
| --- | --- |
| 41.1 | 237 |
| 43 | 159 |
| 55.1 | 255 |
| 55.9 | 142 |
| 68.9 | 197 |
| 77 | 139 |
| 83 | 222 |
| 84 | 473 |
| 91 | 166 |
| 109 | 510 |

Collision Energy

0

Ionization Mode

EI

**Peak List**

| **m/z** | **z** | **Abund** |
| --- | --- | --- |
| 41.1 |  | 503 |
| 42.1 |  | 155 |
| 43.1 |  | 216 |
| 55 |  | 351 |
| 57 |  | 803 |
| 67 |  | 1143 |
| 82 | 1 | 1224 |
| 83 | 1 | 206 |
| 85 |  | 665 |
| 103 |  | 118 |

Collision Energy

0

Ionization Mode

EI

**Peak List**

| **m/z** | **Abund** |
| --- | --- |
| 41.1 | 344 |
| 43.1 | 316 |
| 55 | 352 |
| 56 | 283 |
| 57 | 209 |
| 69 | 264 |
| 83 | 254 |
| 84 | 646 |
| 85 | 280 |
| 109 | 265 |

Collision Energy

0

Ionization Mode

EI

**Peak List**

| **m/z** | **z** | **Abund** |
| --- | --- | --- |
| 41 |  | 405 |
| 43 | 1 | 1445 |
| 53.1 |  | 414 |
| 55 |  | 560 |
| 67 |  | 955 |
| 68 |  | 430 |
| 79 |  | 445 |
| 85 |  | 757 |
| 95 |  | 541 |
| 123 |  | 615 |

Collision Energy

0

Ionization Mode

EI

**Peak List**

| **m/z** | **Abund** |
| --- | --- |
| 41 | 253 |
| 43 | 320 |
| 55 | 250 |
| 69.1 | 585 |
| 77 | 157 |
| 84 | 231 |
| 91 | 257 |
| 94.9 | 198 |
| 106.9 | 373 |
| 179.1 | 699 |

Collision Energy

0

Ionization Mode

EI

**Peak List**

| **m/z** | **z** | **Abund** |
| --- | --- | --- |
| 41 |  | 395 |
| 43 |  | 491 |
| 55 |  | 464 |
| 69 |  | 768 |
| 77 |  | 242 |
| 91 |  | 383 |
| 95 |  | 322 |
| 107.1 |  | 416 |

| **m/z** | **z** | **Abund** |
| --- | --- | --- |
| 179.1 | 1 | 2094 |
| 180.1 | 1 | 297 |

Collision Energy

0

Ionization Mode

EI

**Peak List**

| **m/z** | **z** | **Abund** |
| --- | --- | --- |
| 41.1 |  | 569 |
| 43.1 | 1 | 1992 |
| 67 |  | 561 |
| 68.1 |  | 527 |
| 79 |  | 812 |
| 93.1 |  | 1367 |
| 94.1 |  | 571 |
| 107.1 | 1 | 1263 |
| 121.1 |  | 1072 |
| 136.1 | 1 | 908 |

Collision Energy

0

Ionization Mode

EI

**Peak List**

| **m/z** | **Abund** |
| --- | --- |
| 43 | 585 |
| 54.9 | 229 |
| 77 | 249 |
| 79 | 265 |
| 84 | 578 |
| 91 | 375 |

| **m/z** | **Abund** |
| --- | --- |
| 109 | 373 |
| 119 | 441 |
| 134 | 231 |
| 152.1 | 246 |

Collision Energy

0

Ionization Mode

EI

**Peak List**

| **m/z** | **z** | **Abund** |
| --- | --- | --- |
| 41 |  | 1154 |
| 53 |  | 1062 |
| 67 |  | 1786 |
| 68.1 |  | 1458 |
| 79 |  | 1768 |
| 80 |  | 1400 |
| 81.1 | 1 | 3367 |
| 91 | 1 | 1202 |
| 93 |  | 1951 |
| 107 |  | 1121 |

Collision Energy

0

Ionization Mode

EI

**Peak List**

| **m/z** | **z** | **Abund** |
| --- | --- | --- |
| 41.1 |  | 380 |
| 55 |  | 513 |
| 67 |  | 419 |
| 77 | 1 | 486 |

| **m/z** | **z** | **Abund** |
| --- | --- | --- |
| 79 | 1 | 848 |
| 91.1 |  | 518 |
| 93 |  | 477 |
| 110.1 |  | 459 |
| 149.1 |  | 400 |
| 164.1 |  | 509 |

Collision Energy

0

Ionization Mode

EI

**Peak List**

| **m/z** | **Abund** |
| --- | --- |
| 42.9 | 136 |
| 55 | 134 |
| 79 | 146 |
| 91 | 221 |
| 93 | 124 |
| 105 | 141 |
| 107 | 266 |
| 119 | 142 |
| 121 | 212 |
| 136 | 132 |

Collision Energy

0

Ionization Mode

EI

**Peak List**

| **m/z** | **Abund** |
| --- | --- |
| 41.1 | 314 |
| 55 | 199 |

| **m/z** | **Abund** |
| --- | --- |
| 69.1 | 254 |
| 77 | 240 |
| 79 | 342 |
| 90.9 | 287 |
| 93 | 410 |
| 105 | 189 |
| 107 | 213 |
| 133 | 234 |

Collision Energy

0

**Peak List**

Collision Energy

| **m/z** | **z** | **Abund** |
| --- | --- | --- |
| 41 |  | 315 |
| 76.9 |  | 335 |
| 79 |  | 390 |
| 81 |  | 319 |
| 91 | 1 | 592 |
| 93 | 1 | 357 |
| 105 | 1 | 592 |
| 119 |  | 287 |
| 120 |  | 263 |
| 161.1 | 1 | 1254 |

0

Ionization Mode

EI

Ionization Mode

EI

# Peak List

| **m/z** | **Abund** |
| --- | --- |
| 41.1 | 366 |
| 43 | 144 |
| 52.9 | 140 |
| 55 | 189 |
| 67.1 | 201 |
| 69 | 708 |
| 79 | 165 |
| 81 | 164 |
| 91 | 150 |
| 93 | 367 |

Collision Energy

0

**Peak List**

Collision Energy

| **m/z** | **z** | **Abund** |
| --- | --- | --- |
| 41.1 |  | 531 |
| 55.1 |  | 347 |
| 77 |  | 587 |
| 79 | 1 | 617 |
| 81 |  | 694 |
| 91 | 1 | 920 |
| 93 | 1 | 566 |
| 105 | 1 | 1056 |
| 119.1 |  | 528 |
| 161.1 | 1 | 1366 |

0

Ionization Mode

EI

Ionization Mode

EI

**Peak List**

| **m/z** | **z** | **Abund** |
| --- | --- | --- |
| 41.1 |  | 361 |
| 55.1 |  | 259 |
| 66.9 |  | 360 |
| 77 |  | 283 |
| 79 |  | 371 |
| 80 |  | 637 |
| 91.1 |  | 383 |
| 92 |  | 309 |
| 93 | 1 | 1632 |
| 121 | 1 | 422 |

Collision Energy

0

Ionization Mode

EI

**Peak List**

| **m/z** | **z** | **Abund** |
| --- | --- | --- |
| 41 |  | 329 |
| 77 |  | 310 |
| 79 |  | 292 |
| 81.1 |  | 261 |
| 91 |  | 627 |
| 105 |  | 506 |
| 119 |  | 326 |
| 161.1 | 1 | 1575 |
| 162.1 | 1 | 258 |
| 204.1 |  | 240 |

Collision Energy

0

Ionization Mode

EI

**Peak List**

| **m/z** | **z** | **Abund** |
| --- | --- | --- |
| 41 |  | 321 |
| 55 |  | 238 |
| 77 |  | 354 |
| 79 |  | 380 |
| 81 |  | 324 |
| 91 | 1 | 522 |
| 93 | 1 | 246 |
| 105 |  | 455 |
| 119 |  | 312 |
| 161.1 |  | 744 |

Collision Energy

0

Ionization Mode

EI

**Peak List**

| **m/z** | **z** | **Abund** |
| --- | --- | --- |
| 41 |  | 279 |
| 76.9 |  | 295 |
| 79 |  | 399 |
| 81 |  | 287 |
| 91 | 1 | 496 |
| 93.1 |  | 353 |
| 105 |  | 463 |
| 119 |  | 338 |
| 133.1 |  | 219 |
| 161.1 | 1 | 1011 |

Collision Energy

0

Ionization Mode

EI

**Peak List**

| **m/z** | **z** | **Abund** |
| --- | --- | --- |
| 105.1 |  | 203 |
| 115 |  | 243 |
| 128 |  | 417 |
| 129 |  | 445 |
| 130.9 |  | 313 |
| 143 |  | 177 |
| 144 |  | 297 |
| 159.1 | 1 | 3484 |
| 160.1 | 1 | 441 |
| 202.1 |  | 391 |

Collision Energy

0

Ionization Mode

EI

**Peak List**

| **m/z** | **Abund** |
| --- | --- |
| 41.1 | 121 |
| 55 | 128 |
| 137 | 101 |
| 207.1 | 102 |

Collision Energy

0

Ionization Mode

EI

**Peak List**

| **m/z** | **Abund** |
| --- | --- |
| 41 | 107 |
| 81 | 137 |
| 91 | 149 |
| 93.1 | 113 |
| 105 | 360 |
| 161 | 201 |

Collision Energy

0

Ionization Mode

EI

**Peak List**

| **m/z** | **Abund** |
| --- | --- |
| 41 | 106 |
| 43 | 233 |
| 55.1 | 104 |
| 84 | 193 |
| 91.1 | 161 |
| 91.9 | 108 |
| 92.9 | 116 |
| 109 | 220 |
| 119 | 176 |
| 152.1 | 118 |

Collision Energy

0

Ionization Mode

EI

**Peak List**

| **m/z** | **Abund** |
| --- | --- |
| 43 | 747 |
| 55 | 255 |
| 77 | 297 |
| 83.9 | 749 |
| 91 | 463 |
| 92 | 230 |
| 109 | 436 |
| 119 | 589 |
| 134 | 343 |
| 152. | 380 |

Collision Energy

0

Ionization Mode

EI

**Peak List**

| **m/z** | **z** | **Abund** |
| --- | --- | --- |
| 41 |  | 1362 |
| 53.1 |  | 1193 |
| 67.1 |  | 2098 |
| 68 |  | 1905 |
| 79.1 |  | 2097 |
| 80.1 |  | 1250 |
| 81.1 | 1 | 3408 |
| 91 | 1 | 1388 |
| 93.1 |  | 2325 |
| 107.1 |  | 1380 |

Collision Energy

0

Ionization Mode

EI

**Peak List**

| **m/z** | **z** | **Abund** |
| --- | --- | --- |
| 41.1 |  | 380 |
| 55 |  | 513 |
| 67 |  | 419 |
| 77 | 1 | 486 |
| 79 | 1 | 848 |
| 91.1 |  | 518 |
| 93 |  | 477 |
| 110.1 |  | 459 |
| 149.1 |  | 400 |
| 164.1 |  | 509 |

Collision Energy

0

Ionization Mode

EI

**Peak List**

| **m/z** | **Abund** |
| --- | --- |
| 41 | 151 |
| 43 | 195 |
| 71 | 158 |
| 79 | 160 |
| 91 | 292 |
| 93 | 177 |
| 104.8 | 190 |
| 107 | 347 |
| 119.1 | 205 |
| 121.1 | 229 |

Collision Energy

0

Ionization Mode

EI

**Peak List**

| **m/z** | **Abund** |
| --- | --- |
| 41 | 362 |
| 55 | 241 |
| 66.9 | 221 |
| 68.9 | 296 |
| 77 | 199 |
| 79 | 353 |
| 81 | 199 |
| 91 | 323 |
| 93 | 361 |
| 133.1 | 300 |

Collision Energy

0

Ionization Mode

EI

**Peak List**

| **m/z** | **z** | **Abund** |
| --- | --- | --- |
| 41 |  | 315 |
| 76.9 |  | 335 |
| 79 |  | 390 |
| 81 |  | 319 |
| 91 | 1 | 592 |
| 93 | 1 | 357 |
| 105 | 1 | 592 |
| 119 |  | 287 |

| **m/z** | **z** | **Abund** |
| --- | --- | --- |
| 120 |  | 263 |
| 161.1 | 1 | 1254 |

Collision Energy

0

Ionization Mode

EI

**Peak List**

| **m/z** | **Abund** |
| --- | --- |
| 41 | 627 |
| 52.9 | 203 |
| 55 | 255 |
| 66.9 | 327 |
| 69 | 1118 |
| 79 | 292 |
| 81 | 260 |
| 91 | 215 |
| 93 | 645 |
| 133 | 211 |

Collision Energy

0

Ionization Mode

EI

**Peak List**

| **m/z** | **z** | **Abund** |
| --- | --- | --- |
| 41 |  | 555 |
| 55 |  | 382 |
| 77 |  | 622 |
| 79 | 1 | 644 |
| 81 |  | 675 |
| 91 | 1 | 951 |

| **m/z** | **z** | **Abund** |
| --- | --- | --- |
| 93 | 1 | 604 |
| 105 | 1 | 1003 |
| 119 |  | 557 |
| 161.1 | 1 | 1329 |

Collision Energy

0

Ionization Mode

EI

**Peak List**

| **m/z** | **z** | **Abund** |
| --- | --- | --- |
| 41.1 |  | 288 |
| 55 |  | 237 |
| 67 |  | 294 |
| 77 |  | 272 |
| 79.1 |  | 344 |
| 80 |  | 577 |
| 90.9 |  | 328 |
| 91.9 |  | 244 |
| 93.1 | 1 | 1352 |
| 121 | 1 | 376 |

Collision Energy

0

Ionization Mode

EI

**Peak List**

| **m/z** | **z** | **Abund** |
| --- | --- | --- |
| 41 |  | 321 |
| 55 |  | 238 |
| 77 |  | 354 |
| 79 |  | 380 |

| **m/z** | **z** | **Abund** |
| --- | --- | --- |
| 81 |  | 324 |
| 91 | 1 | 522 |
| 93 | 1 | 246 |
| 105 |  | 455 |
| 119 |  | 312 |
| 161.1 |  | 744 |

Collision Energy

0

Ionization Mode

EI

**Peak List**

| **m/z** | **z** | **Abund** |
| --- | --- | --- |
| 41.1 |  | 278 |
| 55 |  | 215 |
| 77 |  | 241 |
| 79 |  | 307 |
| 81.1 |  | 197 |
| 90.9 |  | 368 |
| 93 |  | 308 |
| 105 |  | 394 |
| 119 |  | 314 |
| 161.1 | 1 | 784 |

Collision Energy

0

Ionization Mode

EI

**Peak List**

| **m/z** | **z** | **Abund** |
| --- | --- | --- |
| 104.9 |  | 182 |
| 115 |  | 230 |

| **m/z** | **z** | **Abund** |
| --- | --- | --- |
| 127.9 |  | 402 |
| 129 |  | 421 |
| 131.1 |  | 297 |
| 143.1 |  | 207 |
| 144 |  | 329 |
| 159.1 | 1 | 3374 |
| 160.1 | 1 | 421 |
| 202.1 |  | 349 |

Collision Energy

0

**Peak List**

| **m/z** | **Abund** |
| --- | --- |
| 41 | 105 |
| 42.9 | 117 |
| 55.1 | 115 |
| 69 | 125 |
| 206.9 | 115 |

Collision Energy

0

Ionization Mode

EI

Ionization Mode

EI

**Peak List**

| **m/z** | **Abund** |
| --- | --- |
| 41.1 | 123 |
| 55.1 | 102 |
| 78.9 | 101 |
| 81.1 | 138 |
| 91 | 141 |

| **m/z** | **Abund** |
| --- | --- |
| 93 | 122 |
| 105 | 422 |
| 161.1 | 214 |

Collision Energy

0

Ionization Mode

EI

**Peak List**

| **m/z** | **Abund** |
| --- | --- |
| 40.9 | 116 |
| 55 | 361 |
| 67 | 346 |
| 82.1 | 387 |
| 83 | 409 |

Collision Energy

0

Ionization Mode

EI

**Peak List**

| **m/z** | **Abund** |
| --- | --- |
| 41 | 117 |
| 43 | 268 |
| 54.9 | 129 |
| 58.1 | 161 |
| 69 | 112 |
| 71 | 126 |

Collision Energy

0

Ionization Mode

EI

**Peak List**

| **m/z** | **Abund** |
| --- | --- |
| 40.9 | 121 |
| 42.9 | 183 |
| 67 | 114 |
| 69 | 244 |
| 81.1 | 118 |

Collision Energy

0

**Peak List**

Collision Energy

| **m/z** | **z** | **Abund** |
| --- | --- | --- |
| 41.1 |  | 471 |
| 43.1 |  | 436 |
| 55.1 |  | 532 |
| 79.1 |  | 410 |
| 91 |  | 362 |
| 95 |  | 480 |
| 105 |  | 511 |
| 119 | 1 | 874 |
| 161.1 | 1 | 840 |
| 179.1 |  | 595 |

0

Ionization Mode

EI

Ionization Mode

EI

**Peak List**

| **m/z** | **z** | **Abund** |
| --- | --- | --- |
| 41.1 |  | 1042 |
| 43.1 |  | 1581 |
| 79.1 | 1 | 1185 |
| 81 | 1 | 1219 |
| 91 |  | 944 |
| 93 |  | 825 |
| 95.1 |  | 1043 |
| 105 | 1 | 1139 |
| 161.1 | 1 | 3579 |
| 204. | 1 | 1379 |

Collision Energy

0

Ionization Mode

EI

**Peak List**

| **m/z** | **z** | **Abund** |
| --- | --- | --- |
| 40.9 |  | 293 |
| 43.1 |  | 632 |
| 71 |  | 311 |
| 79 |  | 374 |
| 81 |  | 272 |
| 91 |  | 268 |
| 92.9 |  | 260 |
| 95 | 1 | 783 |
| 109 |  | 244 |
| 121.1 |  | 561 |

Collision Energy

0

Ionization Mode

EI

**Peak List**

| **m/z** | **Abund** |
| --- | --- |
| 41.1 | 176 |
| 43 | 155 |
| 54.9 | 159 |
| 67 | 188 |
| 69 | 135 |
| 78.9 | 152 |
| 81.1 | 152 |
| 91.1 | 119 |
| 93.1 | 134 |
| 95 | 135 |

Collision Energy

0

Ionization Mode

EI

**Peak List**

| **m/z** | **Abund** |
| --- | --- |
| 41 | 198 |
| 43 | 158 |
| 54.9 | 164 |
| 67 | 130 |
| 69.1 | 126 |
| 77 | 125 |
| 79 | 167 |
| 91 | 175 |
| 93 | 179 |
| 95 | 110 |

Collision Energy

0

Ionization Mode

EI

**Peak List**

| **m/z** | **Abund** |
| --- | --- |
| 41 | 252 |
| 43 | 187 |
| 55 | 220 |
| 67 | 186 |
| 69 | 160 |
| 77 | 189 |
| 79. | 242 |
| 81.1 | 158 |
| 91 | 252 |
| 109.1 | 241 |

Collision Energy

0

Ionization Mode

EI

**Peak List**

| **m/z** | **Abund** |
| --- | --- |
| 41 | 104 |
| 43 | 116 |
| 54.9 | 126 |
| 97 | 132 |

Collision Energy

0

Ionization Mode

EI

**Peak List**

| **m/z** | **Abund** |
| --- | --- |
| 55 | 111 |

Collision Energy

0

Ionization Mode

EI

**Peak List**

| **m/z** | **Abund** |
| --- | --- |
| 40.9 | 101 |
| 93 | 141 |

Spectrum Source

Peak (11) in "+ TIC Scan"

Collision Energy

0

Ionization Mode

EI

**Peak List**

| **m/z** | **z** | **Abund** |
| --- | --- | --- |
| 53 |  | 4062 |
| 67.1 |  | 13043 |

| **m/z** | **z** | **Abund** |
| --- | --- | --- |
| 68.1 | 1 | 16795 |
| 77 |  | 3425 |
| 79 |  | 5818 |
| 91 |  | 3446 |
| 92 |  | 3328 |
| 93.1 |  | 10395 |
| 94.1 |  | 3839 |
| 121.1 |  | 3110 |

Spectrum Source

Peak (29) in "+ TIC Scan"

**Peak List**

| **m/z** | **z** | **Abund** |
| --- | --- | --- |
| 41.1 |  | 15036 |
| 53.1 |  | 19439 |
| 54.1 |  | 50255 |
| 79 |  | 17711 |
| 82 | 1 | 127834 |
| 91 |  | 15434 |
| 93.1 | 1 | 39360 |
| 106.1 |  | 16234 |
| 107.1 |  | 23295 |
| 108.1 |  | 37626 |

Spectrum Source

+ TIC Scan

Collision Energy

0

Collision Energy

0

Ionization Mode

EI

Ionization Mode

EI

# Peak List

| **m/z** | **z** | **Abund** |
| --- | --- | --- |
| 53 |  | 534 |
| 77 |  | 1079 |
| 79 |  | 2328 |
| 80 |  | 4664 |
| 81.1 | 1 | 6166 |
| 91 |  | 980 |
| 105 |  | 726 |
| 123.1 | 1 | 3681 |
| 124.1 | 1 | 486 |
| 161.1 |  | 1495 |

Spectrum Source

Peak (42) in "+ TIC Scan"

**Peak List**

| **m/z** | **z** | **Abund** |
| --- | --- | --- |
| 41.1 | 1 | 2843 |
| 67 |  | 1755 |
| 69.1 | 1 | 3361 |
| 77 |  | 1862 |
| 79 |  | 3036 |
| 81 |  | 1491 |
| 91 |  | 3038 |
| 93.1 | 1 | 3532 |
| 105 |  | 1840 |
| 133.1 | 1 | 2565 |

Spectrum Source

Peak (49) in "+ TIC Scan"

Collision Energy

0

Collision Energy

0

Ionization Mode

EI

Ionization Mode

EI

**Peak List**

| **m/z** | **z** | **Abund** |
| --- | --- | --- |
| 41.1 |  | 564 |
| 55.1 |  | 387 |
| 77 |  | 646 |
| 79 |  | 720 |
| 81 |  | 705 |
| 91 | 1 | 1053 |
| 93 | 1 | 402 |
| 105 | 1 | 1009 |
| 120.1 |  | 425 |
| 161. | 1 | 1538 |

Spectrum Source

+ TIC Scan

Collision Energy

0

Ionization Mode

EI

**Peak List**

| **m/z** | **z** | **Abund** |
| --- | --- | --- |
| 41.1 |  | 672 |
| 43.1 |  | 768 |
| 55 |  | 391 |
| 66.9 |  | 400 |
| 69 |  | 546 |
| 77 |  | 404 |
| 79 | 1 | 834 |
| 91 |  | 392 |
| 93 |  | 581 |
| 95.1 |  | 395 |

Spectrum Source

Peak (56) in "+ TIC Scan"

Collision Energy

0

Ionization Mode

EI

**Peak List**

| **m/z** | **z** | **Abund** |
| --- | --- | --- |
| 41 |  | 425 |
| 43 |  | 614 |
| 69.1 |  | 310 |
| 79 |  | 457 |
| 81 |  | 451 |
| 91 |  | 330 |
| 95 |  | 361 |
| 105 |  | 464 |
| 161.1 | 1 | 1363 |
| 204.1 |  | 504 |

Collision Energy

0

Ionization Mode

EI

**Peak List**

| **m/z** | **Abund** |
| --- | --- |
| 41 | 114 |
| 55 | 101 |

Collision Energy

0

Ionization Mode

EI

**Peak List**

| **m/z** | **Abund** |
| --- | --- |
| 55.1 | 103 |
| 81 | 127 |

Collision Energy

0

Ionization Mode

EI

**Peak List**

| **m/z** | **Abund** |
| --- | --- |
| 41.1 | 156 |
| 55.1 | 104 |
| 66.9 | 130 |
| 77 | 314 |
| 79 | 267 |
| 80.1 | 110 |
| 91 | 396 |
| 92 | 335 |
| 93 | 892 |

Collision Energy

0

Ionization Mode

EI

**Peak List**

| **m/z** | **Abund** |
| --- | --- |
| 41.1 | 160 |
| 77 | 285 |
| 79 | 197 |
| 91 | 299 |
| 93.1 | 682 |

Collision Energy

0

**Peak List**

Collision Energy

| **m/z** | **z** | **Abund** |
| --- | --- | --- |
| 41.1 |  | 501 |
| 67.1 |  | 188 |
| 69 |  | 588 |
| 77 |  | 395 |
| 79 |  | 434 |
| 80.1 |  | 212 |
| 91 |  | 464 |
| 93 | 1 | 1561 |
| 94.1 | 1 | 199 |
| 120.9 |  | 168 |

0

Ionization Mode

EI

Ionization Mode

EI

**Peak List**

| **m/z** | **z** | **Abund** |
| --- | --- | --- |
| 41.1 |  | 605 |
| 53 |  | 131 |
| 67.1 |  | 161 |
| 69.1 |  | 717 |
| 76.9 |  | 143 |
| 79.1 |  | 160 |
| 91 |  | 197 |
| 93 | 1 | 771 |
| 94 | 1 | 101 |

Collision Energy

0

Ionization Mode

EI

**Peak List**

| **m/z** | **z** | **Abund** |
| --- | --- | --- |
| 41.1 |  | 496 |
| 43.1 |  | 306 |
| 43.9 |  | 182 |
| 55 | 1 | 1115 |
| 56.1 | 1 | 120 |
| 57 |  | 295 |
| 57.9 |  | 193 |
| 59 |  | 2134 |
| 83 | 1 | 1074 |
| 101.1 |  | 445 |

Collision Energy

0

Ionization Mode

EI

**Peak List**

| **m/z** | **z** | **Abund** |
| --- | --- | --- |
| 41.1 |  | 541 |
| 43.1 |  | 1065 |
| 55 | 1 | 502 |
| 67 |  | 534 |
| 71.1 |  | 521 |
| 79 |  | 598 |
| 81 | 1 | 763 |
| 91 |  | 588 |
| 93.1 | 1 | 1449 |
| 108 |  | 474 |

Collision Energy

0

Ionization Mode

EI

**Peak List**

| **m/z** | **Abund** |
| --- | --- |
| 41.1 | 163 |
| 53 | 121 |
| 55.1 | 126 |
| 67 | 111 |
| 77 | 244 |
| 79 | 307 |
| 80 | 226 |
| 90.9 | 299 |
| 92 | 133 |
| 93 | 587 |

Collision Energy

0

Ionization Mode

EI

**Peak List**

| **m/z** | **Abund** |
| --- | --- |
| 55 | 218 |
| 59 | 236 |
| 97 | 133 |

Collision Energy

0

Ionization Mode

EI

**Peak List**

| **m/z** | **Abund** |
| --- | --- |
| 41 | 205 |
| 42.9 | 246 |
| 55 | 216 |
| 57 | 369 |
| 70 | 527 |
| 71.1 | 153 |
| 85 | 223 |
| 103 | 103 |

Collision Energy

0

Ionization Mode

EI

**Peak List**

| **m/z** | **Abund** |
| --- | --- |
| 41 | 184 |
| 43 | 205 |
| 44 | 124 |
| 55 | 161 |
| 57 | 380 |
| 70 | 328 |
| 71 | 102 |
| 85 | 188 |

Collision Energy

0

Ionization Mode

EI

**Peak List**

| **m/z** | **Abund** |
| --- | --- |
| 41 | 164 |
| 43.1 | 1114 |
| 55 | 143 |
| 56 | 119 |
| 57.1 | 124 |
| 70 | 127 |
| 83 | 146 |
| 101 | 203 |
| 112.1 | 107 |

Collision Energy

0

Ionization Mode

EI

**Peak List**

| **m/z** | **Abund** |
| --- | --- |
| 42.9 | 133 |
| 79 | 107 |

Collision Energy

0

**Peak List**

| **m/z** | **Abund** |
| --- | --- |
| 41 | 114 |
| 43 | 184 |
| 57.1 | 127 |
| 66.9 | 146 |

Collision Energy

0

Ionization Mode

EI

Ionization Mode

EI

# Peak List

| **m/z** | **Abund** |
| --- | --- |
| 70.9 | 130 |

Collision Energy

0

Ionization Mode

EI

Collision Energy

0

**Peak List**

| **m/z** | **Abund** |
| --- | --- |
| 43 | 105 |
| 59 | 242 |
| 81 | 104 |

Collision Energy

0

Ionization Mode

EI

Ionization Mode

EI

**Peak List**

| **m/z** | **z** | **Abund** |
| --- | --- | --- |
| 41.1 |  | 304 |
| 43 |  | 297 |
| 55.1 |  | 271 |
| 67.1 |  | 298 |
| 79 |  | 437 |
| 81 |  | 246 |
| 93 |  | 372 |
| 107 | 1 | 372 |
| 121 |  | 398 |
| 136 |  | 264 |

Collision Energy

0

Ionization Mode

EI

**Peak List**

| **m/z** | **z** | **Abund** |
| --- | --- | --- |
| 41.1 | 1 | 512 |
| 53 |  | 287 |
| 55.1 |  | 476 |
| 67 |  | 1266 |
| 68.1 |  | 676 |
| 69 | 1 | 447 |
| 81 |  | 545 |
| 82 |  | 558 |
| 95 | 1 | 798 |
| 109.1 |  | 345 |

Collision Energy

0

Ionization Mode

EI

**Peak List**

| **m/z** | **Abund** |
| --- | --- |
| 66.9 | 100 |

Collision Energy

0

Ionization Mode

EI

**Peak List**

| **m/z** | **Abund** |
| --- | --- |
| 41 | 132 |
| 43 | 110 |
| 55 | 151 |
| 66.9 | 101 |
| 68.9 | 116 |
| 83.1 | 151 |
| 84 | 256 |
| 91 | 114 |
| 109 | 308 |

Collision Energy

0

Ionization Mode

EI

**Peak List**

| **m/z** | **Abund** |
| --- | --- |
| 41.1 | 246 |
| 42.9 | 129 |
| 55 | 178 |
| 57.1 | 327 |
| 67.1 | 514 |
| 82 | 492 |
| 85 | 292 |

Collision Energy

0

Ionization Mode

EI

**Peak List**

| **m/z** | **Abund** |
| --- | --- |
| 41 | 217 |
| 42.9 | 157 |
| 54.9 | 191 |
| 56.2 | 124 |
| 69 | 165 |
| 79 | 108 |
| 83 | 155 |
| 84 | 301 |
| 84.9 | 124 |
| 109 | 160 |

Collision Energy

0

Ionization Mode

EI

**Peak List**

| **m/z** | **Abund** |
| --- | --- |
| 41 | 264 |
| 43 | 767 |
| 53.1 | 240 |
| 55 | 283 |
| 67 | 527 |
| 68.1 | 234 |
| 79 | 220 |
| 85 | 445 |
| 95 | 273 |
| 123 | 316 |

Collision Energy

0

Ionization Mode

EI

**Peak List**

| **m/z** | **Abund** |
| --- | --- |
| 41.1 | 155 |
| 43 | 199 |
| 55.1 | 151 |
| 69.1 | 390 |
| 79.1 | 126 |
| 84 | 154 |
| 90.9 | 153 |
| 95 | 121 |
| 107 | 205 |
| 179.1 | 428 |

Collision Energy

0

Ionization Mode

EI

**Peak List**

| **m/z** | **z** | **Abund** |
| --- | --- | --- |
| 41 |  | 243 |
| 43.1 |  | 287 |
| 55 |  | 302 |
| 69 |  | 470 |
| 90.9 |  | 219 |
| 95.1 |  | 192 |
| 106.9 |  | 225 |
| 111.1 |  | 153 |
| 179.1 | 1 | 1264 |
| 180.1 | 1 | 149 |

Collision Energy

0

Ionization Mode

EI

**Peak List**

| **m/z** | **Abund** |
| --- | --- |
| 55 | 182 |
| 67 | 168 |
| 82.1 | 192 |
| 83 | 200 |

Collision Energy

0

Ionization Mode

EI

**Peak List**

| **m/z** | **z** | **Abund** |
| --- | --- | --- |
| 41 |  | 441 |
| 43 |  | 1345 |
| 55 |  | 348 |
| 66.9 |  | 392 |
| 79 |  | 552 |
| 93.1 |  | 959 |
| 94.1 |  | 373 |
| 107 | 1 | 837 |
| 121.1 |  | 684 |
| 136. |  | 559 |

Collision Energy

0

Ionization Mode

EI

**Peak List**

| **m/z** | **Abund** |
| --- | --- |
| 43 | 146 |
| 108.9 | 100 |

Collision Energy

0

Ionization Mode

EI

**Peak List**

| **m/z** | **Abund** |
| --- | --- |
| 41 | 129 |
| 43 | 313 |
| 55 | 141 |
| 84 | 338 |
| 91 | 259 |
| 91.9 | 132 |
| 109 | 200 |
| 119 | 245 |
| 134.1 | 136 |
| 152. | 149 |

Collision Energy

0

Ionization Mode

EI

**Peak List**

| **m/z** | **z** | **Abund** |
| --- | --- | --- |
| 41 |  | 681 |
| 52.9 |  | 612 |
| 67 |  | 1018 |
| 68.1 |  | 943 |
| 79 |  | 951 |
| 80 |  | 673 |
| 81.1 | 1 | 1735 |
| 91 |  | 711 |
| 93.1 |  | 1029 |
| 107 |  | 674 |

Collision Energy

0

Ionization Mode

EI

**Peak List**

| **m/z** | **Abund** |
| --- | --- |
| 41 | 214 |
| 55 | 265 |
| 67 | 179 |
| 76.9 | 225 |
| 78.9 | 392 |
| 91 | 323 |
| 110 | 221 |
| 135.1 | 175 |
| 149 | 188 |
| 164.1 | 246 |

Collision Energy

0

Ionization Mode

EI

**Peak List**

| **m/z** | **Abund** |
| --- | --- |
| 43.1 | 153 |
| 71 | 126 |
| 79 | 128 |
| 91.1 | 163 |
| 93.1 | 113 |
| 104.9 | 119 |
| 107 | 212 |
| 119 | 138 |
| 121 | 137 |

Collision Energy

0

Ionization Mode

EI

**Peak List**

| **m/z** | **Abund** |
| --- | --- |
| 41 | 233 |
| 69 | 198 |
| 76.9 | 118 |
| 79 | 193 |
| 80.9 | 104 |
| 91 | 235 |
| 93 | 225 |
| 105 | 135 |
| 107.1 | 116 |
| 133 | 169 |

Collision Energy

0

Ionization Mode

EI

**Peak List**

| **m/z** | **z** | **Abund** |
| --- | --- | --- |
| 41 |  | 188 |
| 55 |  | 176 |
| 77 |  | 218 |
| 79 |  | 232 |
| 81 |  | 174 |
| 90.9 |  | 389 |
| 92.9 |  | 196 |
| 105 |  | 355 |
| 119 |  | 193 |

| **m/z** | **z** | **Abund** |
| --- | --- | --- |
| 161.1 | 1 | 708 |

Collision Energy

0

Ionization Mode

EI

**Peak List**

| **m/z** | **Abund** |
| --- | --- |
| 41 | 262 |
| 43 | 101 |
| 55.1 | 122 |
| 67 | 141 |
| 69 | 440 |
| 79 | 110 |
| 81.1 | 118 |
| 93.1 | 217 |

Collision Energy

0

Ionization Mode

EI

**Peak List**

| **m/z** | **Abund** |
| --- | --- |
| 41.1 | 216 |
| 43 | 163 |
| 55 | 170 |
| 77 | 280 |
| 79.1 | 235 |
| 81 | 259 |
| 91 | 396 |
| 92.9 | 242 |
| 105 | 358 |

| **m/z** | **Abund** |
| --- | --- |
| 161.1 | 427 |

Collision Energy

0

Ionization Mode

EI

**Peak List**

| **m/z** | **z** | **Abund** |
| --- | --- | --- |
| 41.1 |  | 173 |
| 54.9 |  | 127 |
| 66.9 |  | 163 |
| 76.9 |  | 165 |
| 78.9 |  | 190 |
| 79.9 |  | 235 |
| 91 |  | 185 |
| 92 |  | 134 |
| 93 | 1 | 810 |
| 121 |  | 203 |

Collision Energy

0

Ionization Mode

EI

**Peak List**

| **m/z** | **z** | **Abund** |
| --- | --- | --- |
| 41 |  | 213 |
| 77 |  | 221 |
| 79 |  | 242 |
| 81.1 |  | 227 |
| 91 |  | 482 |
| 104.9 |  | 340 |
| 119 |  | 240 |

| **m/z** | **z** | **Abund** |
| --- | --- | --- |
| 161.1 | 1 | 1323 |
| 162.1 | 1 | 214 |
| 204.1 |  | 217 |

Collision Energy

0

Ionization Mode

EI

**Peak List**

| **m/z** | **Abund** |
| --- | --- |
| 41.1 | 201 |
| 55.1 | 141 |
| 76.9 | 188 |
| 79 | 217 |
| 81 | 195 |
| 91.1 | 300 |
| 93 | 153 |
| 105 | 239 |
| 119.1 | 137 |
| 161.1 | 385 |

Collision Energy

0

Ionization Mode

EI

**Peak List**

| **m/z** | **Abund** |
| --- | --- |
| 41 | 173 |
| 55.1 | 125 |
| 77.1 | 158 |
| 79.1 | 209 |
| 81 | 120 |

| **m/z** | **Abund** |
| --- | --- |
| 91 | 231 |
| 92.9 | 159 |
| 105 | 253 |
| 119 | 156 |
| 161 | 473 |

Collision Energy

0

Ionization Mode

EI

**Peak List**

| **m/z** | **z** | **Abund** |
| --- | --- | --- |
| 105 |  | 126 |
| 114.9 |  | 148 |
| 128 |  | 246 |
| 129 |  | 273 |
| 131 |  | 220 |
| 143 |  | 131 |
| 144 |  | 188 |
| 159.1 | 1 | 1926 |
| 160.1 | 1 | 217 |
| 202 |  | 213 |

Collision Energy

0

Ionization Mode

EI

**Peak List**

| **m/z** | **Abund** |
| --- | --- |
| 55 | 111 |

Collision Energy

0

Ionization Mode

EI

**Peak List**

| **m/z** | **Abund** |
| --- | --- |
| 41 | 111 |
| 90.9 | 113 |
| 105.1 | 233 |
| 161.1 | 126 |

Collision Energy

0

Ionization Mode

EI

**Peak List**

| **m/z** | **Abund** |
| --- | --- |
| 41.1 | 129 |
| 43 | 280 |
| 55 | 173 |
| 57 | 150 |
| 58 | 185 |
| 71 | 125 |

Collision Energy

0

Ionization Mode

EI

**Peak List**

| **m/z** | **Abund** |
| --- | --- |
| 41 | 109 |
| 43 | 131 |
| 55.1 | 108 |
| 67.1 | 117 |
| 69 | 195 |

Collision Energy

0

**Peak List**

| **m/z** | **Abund** |
| --- | --- |
| 55 | 116 |

Collision Energy

0

Ionization Mode

EI

Ionization Mode

EI

# Peak List

| **m/z** | **Abund** |
| --- | --- |
| 55 | 143 |
| 97 | 129 |

Collision Energy

0

Ionization Mode

EI

**Peak List**

| **m/z** | **Abund** |
| --- | --- |
| 41.1 | 167 |
| 43 | 164 |
| 54.9 | 162 |
| 67 | 168 |
| 76.9 | 141 |
| 79 | 181 |
| 81 | 144 |
| 91 | 195 |
| 107 | 124 |
| 109 | 222 |

Collision Energy

0

Ionization Mode

EI

**Peak List**

| **m/z** | **Abund** |
| --- | --- |
| 41 | 130 |
| 43.1 | 115 |
| 55 | 151 |
| 67.1 | 106 |
| 79 | 123 |

| **m/z** | **Abund** |
| --- | --- |
| 81 | 112 |
| 91.1 | 102 |
| 93 | 116 |

Collision Energy

0

Ionization Mode

EI

**Peak List**

| **m/z** | **Abund** |
| --- | --- |
| 41 | 147 |
| 43 | 131 |
| 54.9 | 141 |
| 66.9 | 168 |
| 69 | 107 |
| 79 | 123 |
| 81.1 | 123 |
| 93 | 109 |
| 94.9 | 112 |

Collision Energy

0

Ionization Mode

EI

**Peak List**

| **m/z** | **Abund** |
| --- | --- |
| 41 | 213 |
| 43.1 | 382 |
| 54.9 | 187 |
| 69.1 | 156 |
| 70.9 | 180 |
| 79.1 | 205 |

| **m/z** | **Abund** |
| --- | --- |
| 81 | 208 |
| 95 | 450 |
| 109 | 166 |
| 121 | 321 |

Collision Energy

0

Ionization Mode

EI

**Peak List**

| **m/z** | **Abund** |
| --- | --- |
| 40.9 | 221 |
| 43 | 272 |
| 55 | 295 |
| 78.9 | 196 |
| 81 | 198 |
| 95.1 | 208 |
| 105 | 218 |
| 119.1 | 436 |
| 161.1 | 417 |
| 179.1 | 285 |

Collision Energy

0

Ionization Mode

EI

--- End Of Report ---
